# Satisfaction with dental care services in Great Britain 1998–2019

**DOI:** 10.1186/s12903-022-02343-7

**Published:** 2022-07-26

**Authors:** Majed Almutairi, Gerry McKenna, Ciaran O’Neill

**Affiliations:** 1 Centre for Public Health, School of Medicine, Dentistry and Biomedical Sciences, Queen’s University Belfast, Belfast, UK; 2 Public Health Department, School of Health Sciences, Saudi Electronic University, Riyadh, Saudi Arabia

**Keywords:** Access to care, Quality, Great Britain, NHS, Policy, Austerity

## Abstract

**Background:**

Satisfaction with dental services can provide valuable insights into aspects of quality including access as well as changes in this over time. In the UK publicly funded dental services are ostensibly delivered by private sector general dental practitioners for whom private patients represent an opportunity cost to the provision of care to public patients. This study examined changes in satisfaction as economic circumstances and policy changed in Britain between 1998 and 2019.

**Methods:**

Data were taken from successive waves of the British Social Attitudes Survey a representative cross-sectional survey of the population between 1998 and 2019. Descriptive statistics and a series of logistic regression analyses were used to examine the relationships between satisfaction and a range of socio-demographic characteristics over time.

**Results:**

37,328 usable responses were extracted from the survey spanning 21 years of data. Over the course of the survey approximately 71% of the sample was very satisfied, satisfied or neither satisfied nor dissatisfied with publicly funded dental services. Satisfaction fell at the outset of the study period but rose following the economic downturn from 2008 which coincided with increased use of publicly funded services. Differences were evident in satisfaction between older versus younger respondents, more affluent versus less affluent respondents and better educated versus less well-educated respondents. Satisfaction did not appear to change in response to policy changes.

**Conclusion:**

Satisfaction is an important outcome of service provision. Policies aiming to improve satisfaction with publicly funded dental care in the UK must take account of the competing demands on dentists’ time from private patients. At times of economic expansion or when supply has been disrupted, these may be particularly acute and require specific interventions to improve access for those who depend on public services.

**Supplementary Information:**

The online version contains supplementary material available at 10.1186/s12903-022-02343-7.

## Introduction

Dentists’ behaviours are motivated by both intrinsic and extrinsic factors [1]. Intrinsic factors may include a sense of duty toward one’s patients, professional pride in the quality of services delivered and clinical autonomy. Extrinsic factors include the financial rewards that arise from the delivery of services and the administrative burden associated with collection of those fees. In the UK, the vast majority of publicly funded primary dental care is delivered by self-employed General Dental Practitioners (GDPs) [2]. As self-employed practitioners, GDPs have the option of treating public and private patients with the opportunity cost of one measured in terms of the rewards from  the other foregone [3]. Patients who meet age, health or means tested criteria are eligible to receive a range of services provided free at the point of use with reimbursement made by the State. Other patients face a significant co-pay for such services (with the State meeting the remainder of the cost) or can choose to be wholly private with no State subsidy for the services received [4].

In this context, changes to the arrangements under which payments are made for publicly funded patients (in terms of fees or the administrative burden), and in broader economic conditions that may affect demand from privately funded patients, have the potential to affect extrinsic rewards and with it the supply of GDP effort across patient groups. This in turn has the potential to effect user access to services and their experience of those services in terms for example of waiting time or consultation duration. In this respect, the patient experience of publicly funded services is inextricably linked to broader economic conditions as well as government policies specific to dentistry.

In Great Britain, following the devolution of health policy to Scotland and Wales in 2001, arrangements under which publicly funded dentistry was provided diverged over time [5]. In England and Wales, changes in reimbursement arrangements in 2006 saw the introduction of capped budgets, an end to patient registration and a system of payments that replaced almost 400 distinct service items with just 3 broad bands of payment. Under these reforms, units of dental activity (UDAs) replaced the more granular fee for service arrangement with UDAs varying based on treatment complexity [5].

The UDA system did not offer precision in aligning clinical activity (and the time it took) with rewards for the dentist providing care. Indeed, in specific instances it served to create perverse incentives where, for example, a dentist would receive the same payment if they extracted a tooth as they would were they to undertake complex and potentially time-consuming endodontic treatment. Unease in the profession around this was compounded by the introduction of capped budgets that served to deter dentists from accepting publicly funded patients with complex needs. The termination of registration simultaneously afforded dentists greater opportunity to avoid such patients. These changes were not enacted in Scotland which ostensibly continued to follow the pre-2006 fee-per-item contract. Registration continued with capitation payments used to encourage wider access. In addition, practice grants were available to encourage investment in facilities while the granular fee for service arrangement allowed payments to more closely reflect effort/cost involved in their provision [6].

The 2006 reforms saw large and abrupt changes in activity by dentists in England with a dramatic fall in the number of more complex procedures provided [7]. Unhappiness among dentists regarding administrative arrangements—which induced anxiety around the financial implications of contract underperformance—were also noted [8]. A review of the reforms by a House of Commons Select Committee in 2008 and an independent review by Steele in 2009 [9], echoed these concerns and pointed to issues with an increase in patient charges and failures to improve access. While UDAs were retained, commissioning arrangements changed again in 2013 in England, in part in recognition of the shortcomings of the previous reforms [10].

Important as these policy changes were, however, they did not happen in isolation but rather within a broader context of profound economic change. The financial crisis of 2008 and the subsequent economic downturn that followed, raised unemployment levels across Britain, increased job insecurity and ushered in a period of austerity in public spending that affected incomes in both the public and private sector. The outworking of this economic shock had the potential to reduce demand for privately funded dental care across Britain and with it the relative attractiveness of public compared with private provision. In short, the first 20 years of twenty-first century saw changes to dentistry in England and Wales mooted in 2004 and adopted from 2006 that were not adopted in Scotland. It saw changes in the broader economic climate across Britain that had the potential to change access, quality, and user experience of publicly funded services. From the perspective of the public, each had the potential to impact their experience of publicly funded dental services and their satisfaction with them. Satisfaction with respect to health care is a poorly defined concept used in different ways across different studies [11–16]. How it should be measured and interpreted remains the subject of debate [17]. That it can be indicative of a services’ success in meeting the expectations of users with respect to the elements of service they deem important, is though generally conceded and various studies have shown satisfaction to be positively correlated with clinical outcomes and service utilization [18–21]. In this paper we examine changes in patient satisfaction over 22 years in Britain and relate this to changes in policy and the broader economic climate to assess trends and predictors of public satisfaction with NHS dental services.

## Materials and methods

Data were gathered from the British Social Attitudes Survey (BSAS) from 1998 to 2019 [22]. BSAS is a repeated cross-sectional survey of public attitudes undertaken annually in Britain. The survey is designed to yield a distinct representative sample of community dwelling adults aged 18 and over. A multistage sampling approach is used to construct the sample based on a representative selection of postcodes, random sampling of addresses within those postcodes and random sampling from among adults aged over 18 within the household [23]. While questions vary each year, depending on the themes to be explored in that year, core socio-demographic questions are repeated each year as are a range of attitudinal questions including those related to satisfaction with various publicly funded health services. Respondents are asked to rate their satisfaction with NHS dental services on a five-point scale that ranges from very satisfied, through satisfied, neither satisfied nor dissatisfied to dissatisfied and very dissatisfied. Options to report “don’t know” are also provided. The precise wording of the question and options are reported in Additional file 1. To facilitate interpretation of results and the conduct of additional analyses, satisfaction was re-defined as a dichotomous variable in which very satisfied, satisfied and neither satisfied nor dissatisfied were coded as one and other levels of satisfaction as zero. Other values were treated as missing.

A range of socio-demographic characteristics were extracted from the survey. These included age (whether respondent was 65 or over), household income (in quartiles), ethnicity (whether the respondent was White) education (whether the respondent had obtained a third level degree); gender (whether the respondent was male), marital status (whether the respondent was married/living as such) and whether the household included dependent children. In each case the comparator group defined the base category, that is, aged less than 65, the lowest quartile of income, non-White background, no degree qualification etc. Income was specified as quartiles for the specific year to which the data related rather than as an actual value. This obviated the need to adjust income for inflation, the quartile providing a measure of relative affluence. The socio-demographic data extracted were informed by previous analyses of satisfaction with health services [11, 24–26]. As the data constituted a repeated cross section, sampling weights for each year were extracted as was the year in which the survey was conducted. Further, as policy changes adopted in England and Wales did not extend to Scotland and additional financial supports were made in Scotland to GDPs that may have affected access [3], whether the respondent resided in Scotland, as opposed to England or Wales based on where the respondent resided ( i.e., where the survey was conducted) was also extracted. As the study involved secondary analysis of an anonymised publicly available dataset, no ethical approval was necessary.

Data were pooled across the 21 years of the survey. Descriptive statistics (proportions together with their associated 95% confidence intervals) were produced for variables used in the analyses. Multivariable logistic regression analyses were undertaken in which satisfaction with dental services was estimated as a function of the socio-demographic characteristics detailed above together with a trend variable for the year of the survey. To allow for the possibility that estimated coefficients may not remain stable over time – for example that the relationship between age (say) and satisfaction may vary over time – analyses were repeated wherein covariates were interacted with the trend variable. Results were reported as predicted odds ratios with confidence intervals. All analyses were conducted on weighted data and repeated on unweighted data. Unweighted results are reported in Additional file 2. All analyses were conducted in Stata version 16.0.

## Results

Sample descriptive statistics are reported in Table 1. As can be seen and taking the period as a whole approximately 71% of the sample were satisfied with NHS dental services. Roughly one third of the sample had dependent children in the household, over 92% were White and approximately 9% of the sample were from Scotland as opposed to England and Wales. While the survey is designed and weighted in such a way as to produce results representative of the population, it should be borne in mind when the data is pooled across years, it may not reflect patterns in a given year. For example, in 2011 in England and Wales about 14% of the population was non-White [27]. In Table 2 the results of a logistic regression in which satisfaction is expressed as a function of the variables shown is presented. As can be seen and taking the period as a whole, those resident in Scotland were significantly more likely to express satisfaction with NHS dental services than those who lived in England or Wales—almost 44% more likely (p < 0.05). Those with higher incomes (quartile 4, 18%, p < 0.01) and who were better educated (23%, p < 0.01) were significantly less likely to express satisfaction with services as were those who were married (14%, p < 0.01) and those who were White (13%, p < 0.01). Satisfaction is seen to vary markedly over time. In 2004 the likelihood of a respondent expressing satisfaction falls relative to that in 1998 and remains significantly lower until 2011 after which it becomes significantly greater for the remainder of the period.

**Table 1:** Sample descriptive statistics

Variable	Mean	Std. Dev
*Percentage of sample*		
Satisfied with dental services	71.56	45.11
*With dependent child in household*		
No	67.11	46.98
Yes	32.89	46.98
*With degree*		
No	81.80	38.58
Yes	18.20	38.58
*Married*		
No	43.77	49.61
Yes	56.23	49.61
*Income quartile*		
1	27.74	44.77
2	24.94	43.27
3	24.17	42.81
4	23.16	42.18
*Race*		
Not white	7.58	26.47
White	92.42	26.47
*Respondents over age 65*		
No	79.85	40.11
Yes	20.15	40.11
*By year*		
1998	7.15	25.76
1999	7.36	26.12
2000	7.75	26.74
2001	4.87	21.53
2002	5.00	21.80
2003	4.89	21.56
2004	7.22	25.89
2005	6.95	25.43
2006	4.69	21.13
2007	6.07	23.87
2008	6.65	24.91
2009	6.82	25.22
2010	6.16	24.04
2011	1.96	13.87
2012	2.03	14.10
2013	2.00	13.98
2014	2.03	14.11
2015	2.21	14.69
2016	1.90	13.64
2017	2.23	14.76
2018	1.98	13.93
2019	2.09	14.31
*Male*		
No	56.16	49.62
Yes	43.85	49.62
*Scotland*		
No	9.08	28.85
Yes	9.17	28.85
Sample size	37,238

**Table 2:** Logistic regression of satisfaction with NHS dental services

Independent variable	Odds ratio	Z− statistic	p− value
Had dependent child in household	1.0932	3.06	< 0.01
Had degree	0.7671	− 7.67	< 0.01
Married	0.8591	− 5.05	< 0.01
*In income quartile (relative to 1)*			
2	0.9299	− 1.87	0.06
3	0.8971	− 2.63	< 0.01
4	0.8264	− 4.33	< 0.01
Male	0.9808	− 0.74	0.46
Resides in Scotland	1.4394	7.67	< 0.01
White	0.8698	− 2.71	< 0.01
Over 65	1.2790	6.58	< 0.01
*Year (relative to 1998)*			
1999	1.0073	0.11	0.92
2000	1.3735	4.44	< 0.01
2001	1.0191	0.24	0.81
2002	1.0634	0.80	0.43
2003	0.9006	− 1.39	0.16
2004	0.5123	− 10.26	< 0.01
2005	0.6075	− 7.51	< 0.01
2006	0.5090	− 9.34	< 0.01
2007	0.6103	− 7.16	< 0.01
2008	0.5729	− 8.35	< 0.01
2009	0.6796	− 5.70	< 0.01
2010	0.8604	− 2.10	0.04
2011	1.1324	1.15	0.25
2012	1.3981	2.91	< 0.01
2013	1.4680	3.39	< 0.01
2014	1.3008	2.35	< 0.01
2015	1.6001	4.16	< 0.01
2016	1.9655	5.50	< 0.01
2017	1.5025	3.67	< 0.01
2018	1.6083	3.89	< 0.01
2019	1.9074	5.30	< 0.01

Wald Chi^2^ = 1161.04 (p < 0.01); N = 37,238

**Fig. 1:**
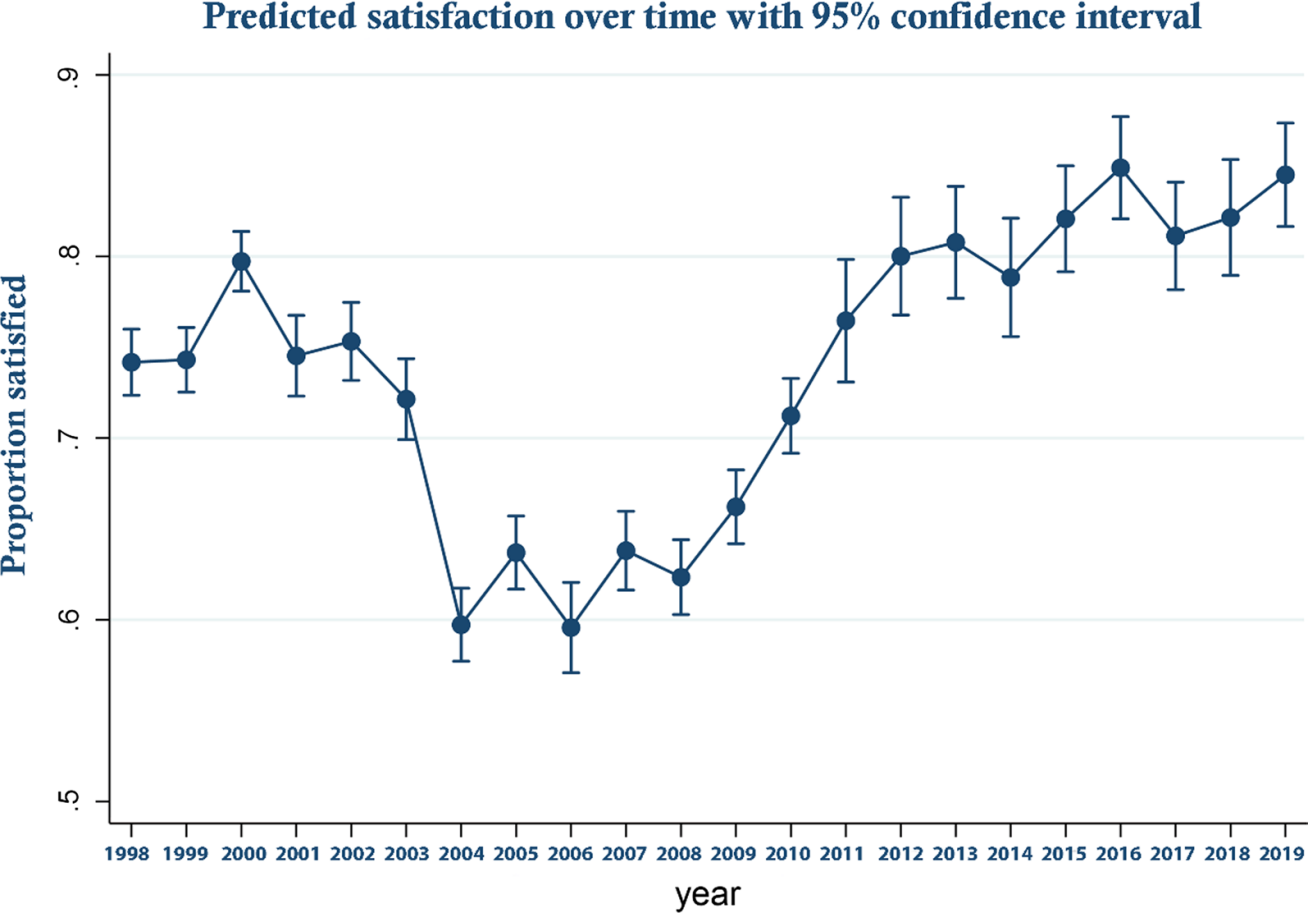
Predicted satisfaction over time

**Fig. 2:**
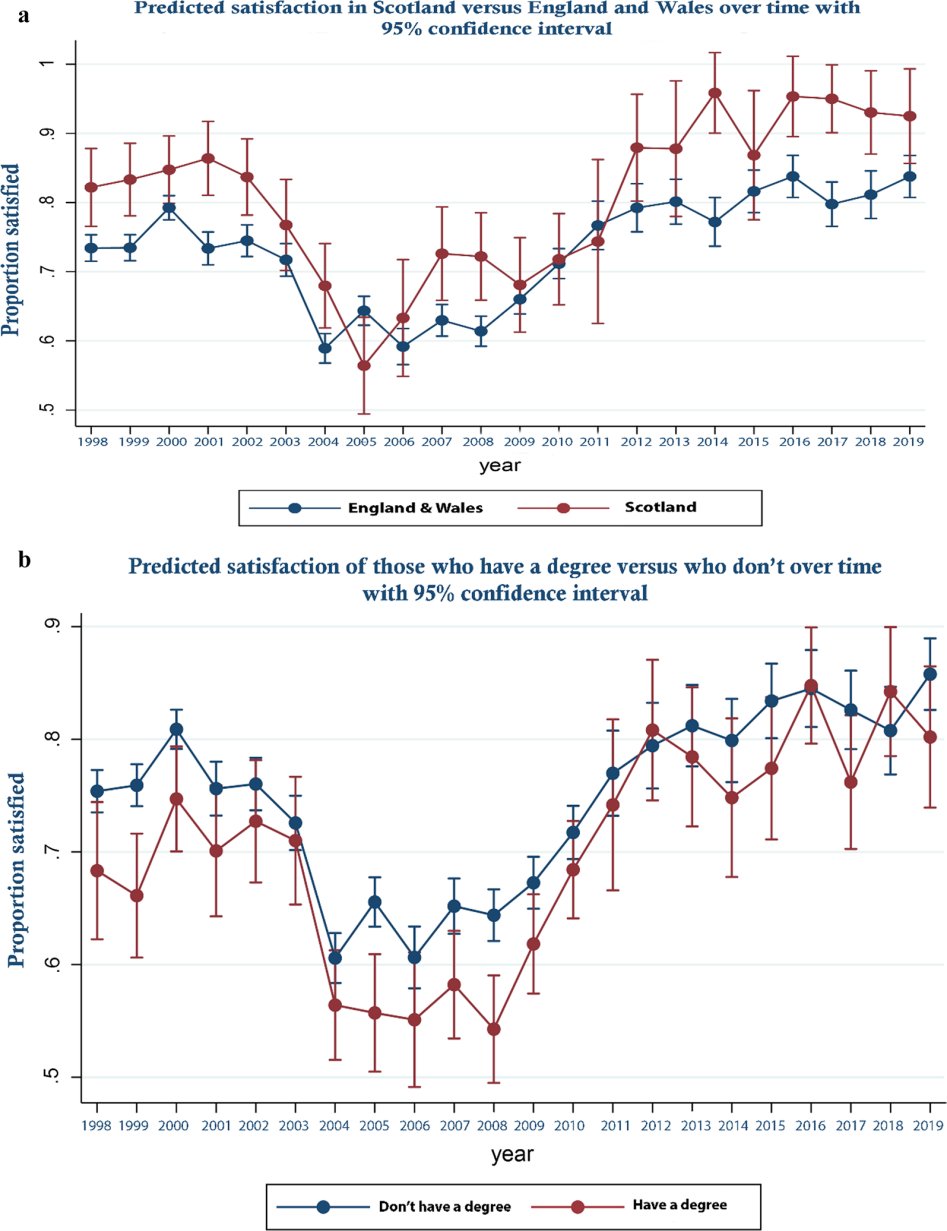

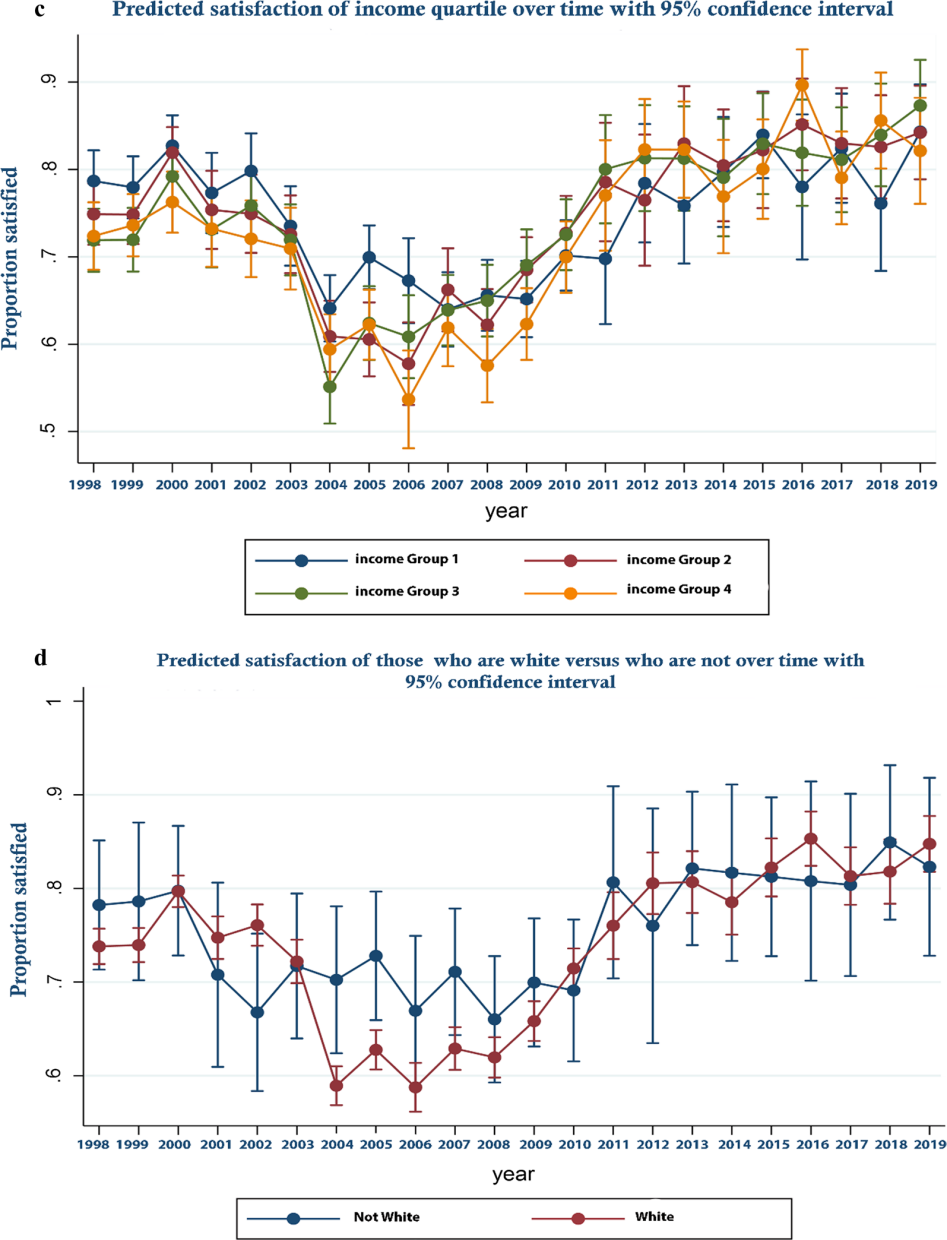
**a** Predicted satisfaction in Scotland versus England and Wales over time. **b** Predicted satisfaction of those who have a degree versus who don’t over time. **c** Predicted satisfaction of income quartile over time. **d** Predicted satisfaction of those who are white versus who are not over time

**Fig. 3:**
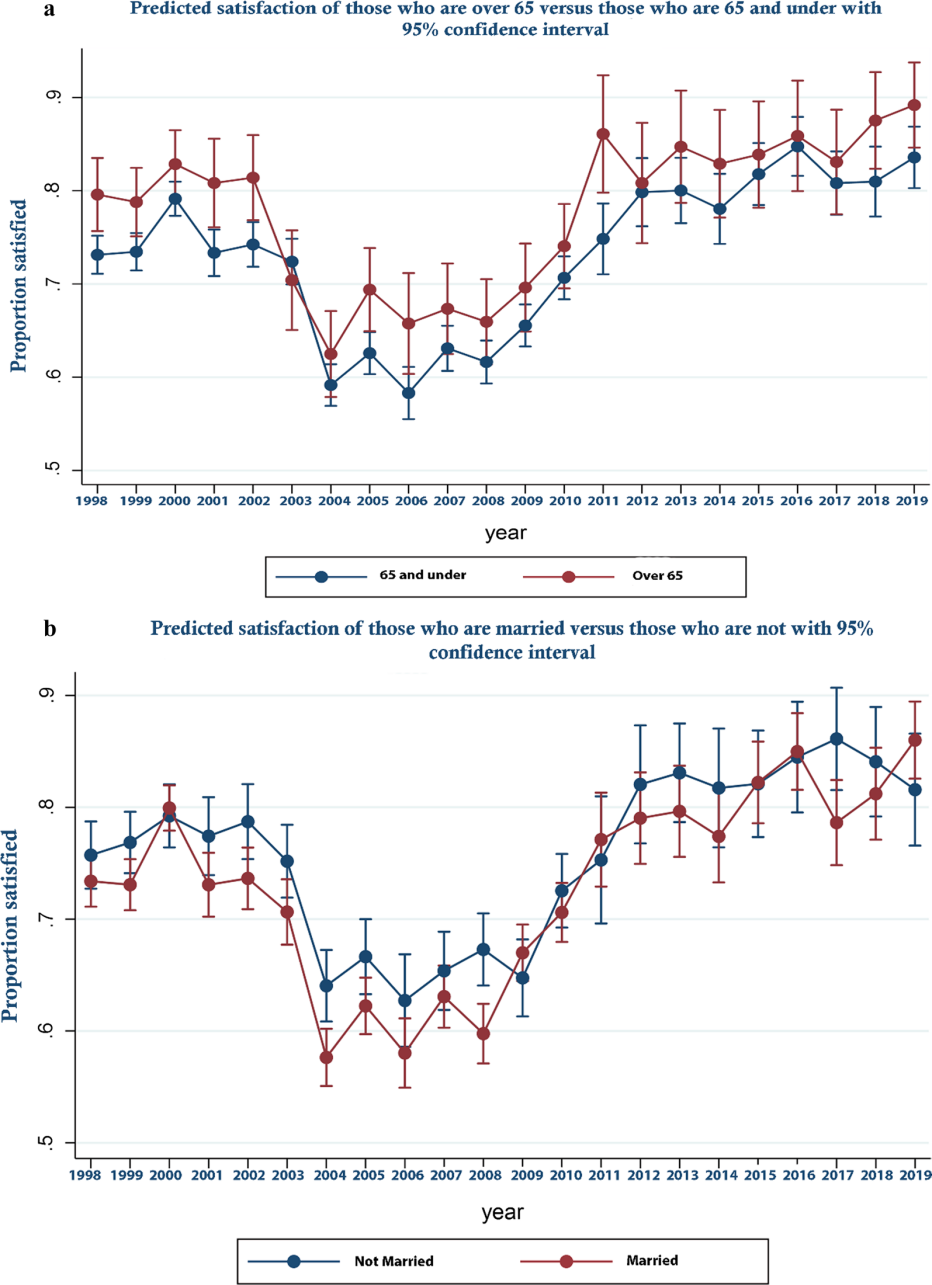

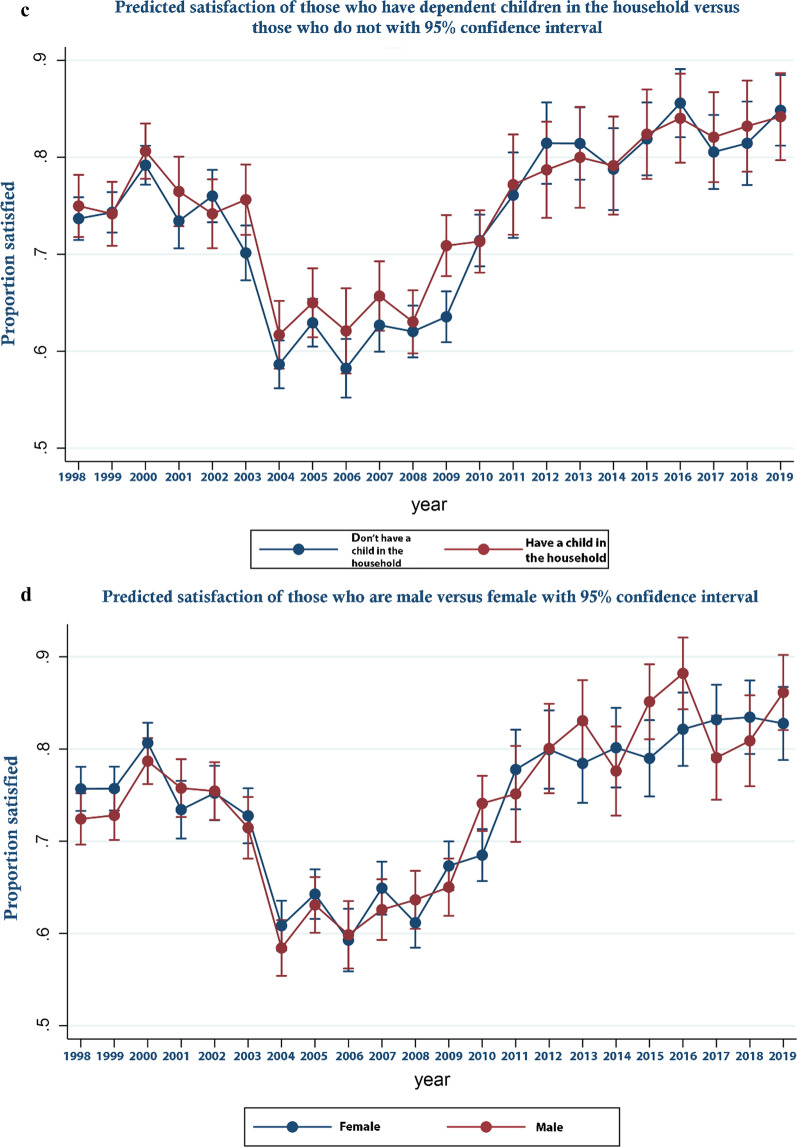
**a** Predicted satisfaction of those who are over 65 versus those who are not. **b** Predicted satisfaction of those who are married versus those who are not. **c** Predicted satisfaction of those who have dependent children in the household versus those who do not. **d** Predicted satisfaction of those who are male versus female

These trends are perhaps more clearly illustrated in Fig. 1 which displays the predicted odds ratios from the logistic regression with respect to year along with their associated 95% confidence intervals—that is predicted satisfaction over time controlling for covariates. As can be seen, satisfaction decreased between 2000 and 2004 becoming significantly lower from 2004 and rising again from 2008 to become significantly higher from 2012 onward. In Fig. 2a–d predicted margins for those from Scotland compared to England and Wales; those who held a degree or above in terms of educational attainment, compared to those who did not; those in income quartile 1 compared to the others; and those who were White compared to those who were not are shown over time. These show relative changes in satisfaction between groups over time. In Fig. 3a–d, similar analyses are presented for those who are married versus those who are not, those who are male versus female, households that had dependent children versus those who did not and who are over 65 versus those who are not are shown. As can be seen, by reference to the failure of confidence intervals to overlap in the Fig. 2a for example, while in general those in Scotland were significantly more likely to be satisfied than those in England and Wales, this was not the case in every year, in 2009 and for a number of subsequent years satisfaction increased more rapidly in England and Wales than it did in Scotland. Similarly, while satisfaction was generally lower for those on higher incomes, those who were better educated, those who were White and those without dependent children, around 2009 satisfaction rose more sharply among these groups than their comparators. Regression results underpinning these figures are presented in Additional file 3. Additional file 4 all analyses are repeated for the unweighted sample. As an additional sensitivity analysis, we repeated the analysis reported in Fig. 1 but assigned those who were neither satisfied nor dissatisfied to the dissatisfied group. The result are report in online supplement 4. The reassignment made no material difference to the trend in satisfaction over time reported in Fig. 1.

## Discussion

Satisfaction studies can provide valuable insights into how well a system meets the needs of users and how the quality of a service might be improved [28]. By extension, they can provide insight into changes in service performance over time. However, any satisfaction data should be interpreted with care given the conceptual and methodological difficulties in its measurement and that they are likely to be related to public and patient expectations which may be mutable over time. Previous studies of satisfaction with dental services in the UK show higher levels of satisfaction with dental services historically than are recorded here [29, 30]. While a previous study has shown satisfaction to vary over time [30], it did not examine satisfaction across groups differentiated by socio-demographic status nor sought to link changes in satisfaction to policy or broader economic changes. A number of studies have reported on satisfaction with health services in general in the UK including dental services [31, 32]^.^ These have tended to show high levels of satisfaction and higher levels among those who are older and those who have had recent contact with services [33]. Among services in general they have shown those with higher incomes and those who are from ethnic minorities are less satisfied [33]. Our results are broadly consistent with these.

Our analyses show that while disquiet was expressed by the profession around policies adopted in England and Wales around public funding of general dental practice that were not adopted in Scotland, public satisfaction in both jurisdictions shifted in a parallel manner at around the time these policies were piloted and adopted, 2004–06. This suggests that while the policies may have impacted on dentists and on the experience of particular patients, among the wider public they appear not to have had a material effect on population satisfaction nor that of specific sub-groups. By contrast the economic downturn that followed the financial crisis of 2008 coincided with a sharp increase in satisfaction among respondents at a time when the policy context remained relatively stable. This is consistent with dentists re-distributing effort toward publicly funded care in a way that may have improved access and hence satisfaction. It is not possible to generate a statistical series distinguishing public and private dental service use in the UK. Statistics for England [34] show that between 2006 and 2016 that while the percentage of the population who saw a NHS dentist fell between 2006 and 2007, between 2008 and 2011 it rose year on year remaining relatively stable between 2013 and 2014 before falling in 2015 back to 2007 levels (after this a series is not available). These changes in use are consistent with an improvement in access from 2008 and (with the exception of 2015) are consistent with the changes in predicted satisfaction reported here. That the increase in satisfaction was sharper among groups one might assume would face significant co-pays for use of public services those who are better educated, higher paid, who do not have dependent children etc.—does not invalidate this. Rather it may reflect a greater willingness/ability of dentists to accept co-paying NHS patients rather than treat them wholly as private patients. If the figures for England are indeed indicative of improved access to publicly funded care in the wake of the financial crisis and did so to a greater extent for those specific groups, it is entirely logical that satisfaction with public services should also increase.

Other results from our study echo those of previous analyses of satisfaction with dental services. Those who are older. Those who are less well-off and those who are less well educated were found to have higher satisfaction levels here and in other studies [28, 29]^.^ This is consistent with expectations given these groups would likely face lower co-pays than those who are younger, more affluent or better educated under the system of entitlement that exists in the United Kingdom. This is similarly the case for those who had dependent children and (given the likely correlation between ethnicity and socio-economic status) for non-Whites.

The analysis has implications for policy makers seeking to improve population oral health through GDP services in dental systems in the UK currently and in systems similar to that of the UK. This is particularly true in the aftermath of the COVID-19 pandemic. While policy makers may obsess about how best to measure and incentivise the supply of effort by dentists to the public system, if they fail to take account of the opportunities provided by private patients, they run the risk of becoming rapidly overtaken by broader economic events that change the calculus of cost and benefit from the perspective of GDPs. In these circumstances, access to care, the sine qua non for other aspects of the user experience, must be considered within the context of opportunity costs facing dentists. Our analysis is consistent with the argument that broader economic changes were associated with changes in access and in public satisfaction independent of policy changes. This is consistent with access changing more in response such economic conditions than policy measures at least from the public’s perspective.

There are a number of limitations to the analysis. First, the data used represent repeated cross-sectional samples of the population rather than a panel dataset and should be interpreted as associations not causal relationships. It is conceivable, for example, that changes in satisfaction were caused by changes in expectations, press reports or other factors rather than the funding environment. Second, and related to this, no data on respondent needs for dental services that may affect their satisfaction was available. Third, no information on actual use of services among respondents whether public or private which may also affect reported satisfaction with NHS services was available. Fourth, while the survey was constructed to provide a representative sample of the population, missing data may have resulted in a less than fully representative sample.

## Conclusions

In Britain publicly funded care is delivered ostensibly by self-employed general dental practitioners for whom private patients provide an alternative source of remuneration to publicly funded patients. Changes in broader economic conditions will likely influence the opportunities presented by private patients for general dental practitioner services and with it the access they are willing and able to provide for publicly funded patients. Our analysis has shown that while policies governing the reimbursement of dentists changed and diverged between constituent parts of Britain, public satisfaction appeared to be more strongly correlated with broader economic conditions than government dental policy. Dental policies that do not incorporate an ability to respond to changes in broader economic conditions could rapidly misalign with the private market for care and see issues with access emerge especially for those dependent on publicly funded care.

## Supplementary Information

**Additional file 1**. Questionnaire wording.

**Additional file 2**. Weighted analysis.

**Additional file 3**. Unweighted analysis.

**Additional file 4.** Weighted analysis in where neither satisfied nor dissatisfied is grouped with dissatisfied groups.

## Data Availability

The data that support the findings of this study are openly available in (UK Data Service) at https://beta.ukdataservice.ac.uk/datacatalogue/studies/study?id=8772, (Doi): http://doi.org/10.5255/UKDA-SN-8772-1, Reference Number (8772).
