# Supplementary material for: Satisfaction with dental care services in Great Britain 1998–2019
Source: BMC Oral Health. 2022 Jul 26;22:308. doi: 10.1186/s12903-022-02343-7 (PMC9315088; doi:10.1186/s12903-022-02343-7)
Supplement: Supplementary file 1 — Additional file 1. Questionnaire wording. [file 12903_2022_2343_MOESM1_ESM.docx]

**Online Supplement 1:**

[DentSat] CARD D5 AGAIN (And how satisfied or dissatisfied are you with the NHS as regards...) ... National Health Service dentists?

1 Very satisfied

2 Quite satisfied

3 Neither satisfied nor dissatisfied

4 Quite dissatisfied

5 Very dissatisfied

8 (Don't know)

9 (Refusal)
