# Supplementary material for: Satisfaction with dental care services in Great Britain 1998–2019
Source: BMC Oral Health. 2022 Jul 26;22:308. doi: 10.1186/s12903-022-02343-7 (PMC9315088; doi:10.1186/s12903-022-02343-7)
Supplement: Supplementary file 2 — Additional file 2. Weighted analysis. [file 12903_2022_2343_MOESM2_ESM.docx]

**Online Supplement 2 :**

**Table S1: Satisfaction with publicly funded dental services as a function of the variables shown ( weighted)**

| **Independent variable** | **Odds Ratio** | **Std. Err.** | **z** | **P>\|z\|** |
| --- | --- | --- | --- | --- |
|  |  |  |  |  |
| **Over65** | **1.2789** | **.0478** | **6.58** | **0.000** |
|  |  |  |  |  |
| **Had Degree** | **.7670** | **.0265** | **-7.67** | **0.000** |
|  |  |  |  |  |
| **Had Dependent Child in Household** | **1.0931** | **.0318** | **3.06** | **0.002** |
|  |  |  |  |  |
| **Married** | **.8591** | **.0258** | **-5.05** | **0.000** |
|  |  |  |  |  |
| **Income Quartile (relative to 1)** |  |  |  |  |
| **2** | **.9298** | **.0362** | **-1.87** | **0.062** |
| **3** | **.8970** | **.0370** | **-2.63** | **0.009** |
| **4** | **.8263** | **.0364** | **-4.33** | **0.000** |
|  |  |  |  |  |
| **Resides in Scotland** | **1.4393** | **.0683** | **7.67** | **0.000** |
|  |  |  |  |  |
| **White** | **.8697** | **.0447** | **-2.71** | **0.007** |
|  |  |  |  |  |
| **Male** | **.9808** | **.0257** | **-0.74** | **0.462** |
|  |  |  |  |  |
| **Year and Resident in England/Wales (relative to 1998 and living in Scotland)** | | | | |
| **2** | **1.0073** | **.0688** | **0.11** | **0.915** |
| **3** | **1.3734** | **.0981** | **4.44** | **0.000** |
| **4** | **1.0190** | **.0790** | **0.24** | **0.807** |
| **5** | **1.0633** | **.0818** | **0.80** | **0.425** |
| **6** | **.9006** | **.06772** | **-1.39** | **0.164** |
| **7** | **.5123** | **.03340** | **-10.26** | **0.000** |
| **8** | **.6074** | **.0403** | **-7.51** | **0.000** |
| **9** | **.5090** | **.0368** | **-9.34** | **0.000** |
| **10** | **.6103** | **.0421** | **-7.16** | **0.000** |
| **11** | **.5728** | **.0382** | **-8.35** | **0.000** |
| **12** | **.6795** | **.0460** | **-5.70** | **0.000** |
| **13** | **.8604** | **.0614** | **-2.10** | **0.035** |
| **14** | **1.1323** | **.1226** | **1.15** | **0.251** |
| **15** | **1.3981** | **.1612** | **2.91** | **0.004** |
| **16** | **1.4680** | **.1663** | **3.39** | **0.001** |
| **17** | **1.3007** | **.1454** | **2.35** | **0.019** |
| **18** | **1.6001** | **.1808** | **4.16** | **0.000** |
| **19** | **1.9655** | **.2415** | **5.50** | **0.000** |
| **20** | **1.5024** | **.1667** | **3.67** | **0.000** |
| **21** | **1.6082** | **.1965** | **3.89** | **0.000** |
| **22** | **1.9074** | **.2324** | **5.30** | **0.000** |
|  |  |  |  |  |
| **_cons** | **3.85845** | **.28717** | **18.14** | **0.000** |

**Wald chi2(31) = 1161.04 (p<0.01) N = 37,238**

**
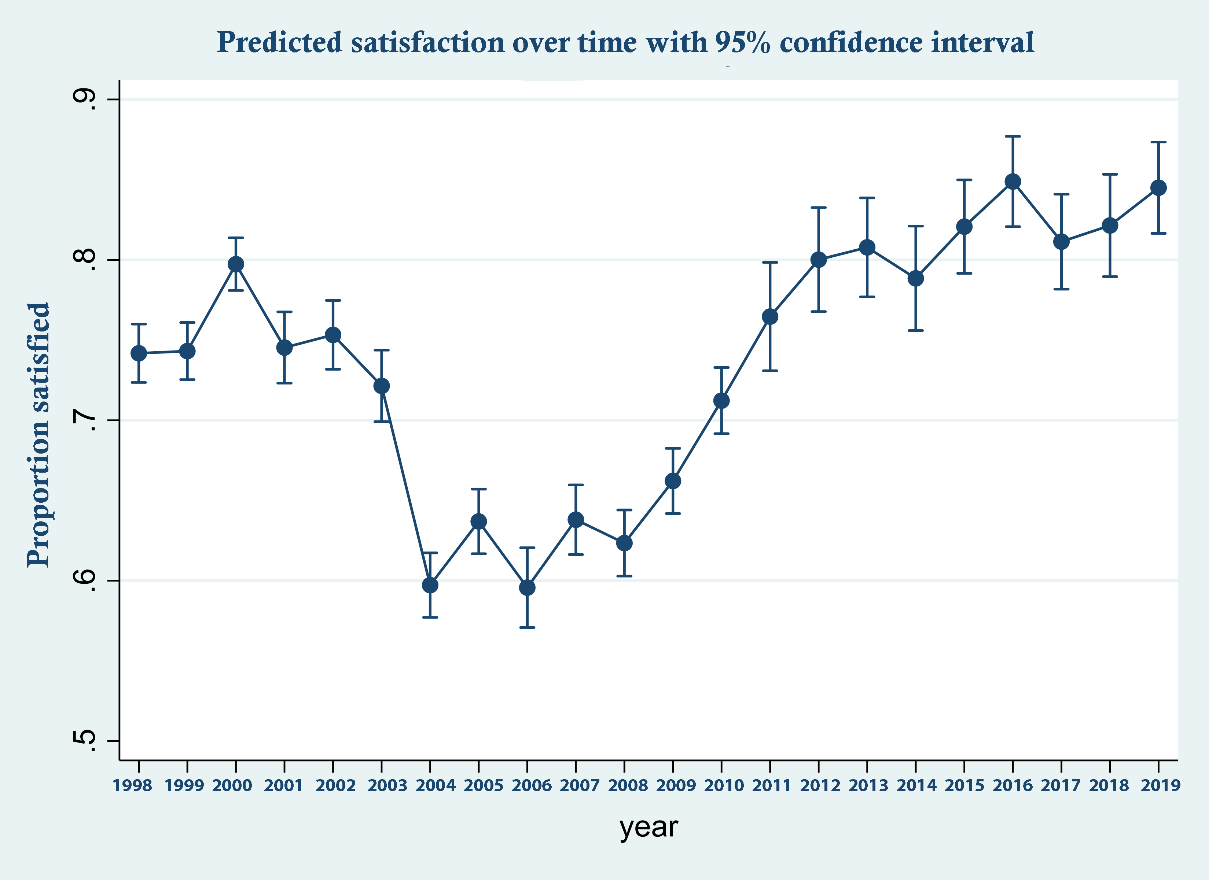
**

**Figure S1: Satisfaction with publicly funded dental services over time.**

**Table S2 Logistic regression of satisfaction with NHS dental services comparing Scotland with England and Wales**

| **Independent variable** | **Odds ratio** | **Z-statistic** | **p-value** |
| --- | --- | --- | --- |
| **Had Dependent Child in Household** | **1.0935** | **3.07** | **<0.01** |
|  |  |  |  |
| **Had Degree** | **0.7656** | **-7.72** | **<0.01** |
|  |  |  |  |
| **Married** | **0.8607** | **-4.98** | **<0.01** |
|  |  |  |  |
| **In Income quartile (relative to 1)** |  |  |  |
| **2** | **0.9296** | **-1.87** | **0.06** |
| **3** | **0.8971** | **-2.62** | **<0.01** |
| **4** | **0.8263** | **-4.32** | **<0.01** |
|  |  |  |  |
| **Male** | **0.9789** | **-0.81** | **0.42** |
|  |  |  |  |
| **Resides in Scotland** | **1.6775** | **2.54** | **<0.01** |
|  |  |  |  |
| **White** | **0.8708** | **-2.69** | **<0.01** |
|  |  |  |  |
| **Over 65** | **1.2786** | **6.57** | **<0.01** |
|  |  |  |  |
| **Year and Resident in England/Wales (relative to 1998 and living in Scotland)** | | | |
| **1999** | **1.0023** | **0.03** | **0.97** |
| **2000** | **1.3861** | **4.41** | **<0.01** |
| **2001** | **0.9971** | **-0.04** | **0.97** |
| **2002** | **1.0574** | **0.70** | **0.49** |
| **2003** | **0.9170** | **-1.11** | **0.27** |
| **2004** | **0.5162** | **-9.75** | **<0.01** |
| **2005** | **0.6510** | **-6.22** | **<0.01** |
| **2006** | **0.5218** | **-8.67** | **<0.01** |
| **2007** | **0.6129** | **-6.84** | **<0.01** |
| **2008** | **0.5729** | **-8.04** | **<0.01** |
| **2009** | **0.7013** | **-5.05** | **<0.01** |
| **2010** | **0.8928** | **-1.53** | **0.13** |
| **2011** | **1.1930** | **1.56** | **0.12** |
| **2012** | **1.3853** | **2.72** | **<0.01** |
| **2013** | **1.4644** | **3.28** | **<0.01** |
| **2014** | **1.2274** | **1.79** | **0.07** |
| **2015** | **1.6133** | **4.10** | **<0.01** |
| **2016** | **1.8781** | **5.01** | **<0.01** |
| **2017** | **1.4311** | **3.14** | **<0.01** |
| **2018** | **1.5620** | **3.54** | **<0.01** |
| **2019** | **1.8766** | **5.02** | **<0.01** |
| **Year and Resident in Scotland (relative to 1998 and living in England/Wales)** | | | |
| **1999** | **1.0825** | **0.29** | **0.774** |
| **2000** | **1.2047** | **0.67** | **0.500** |
| **2001** | **1.3772** | **1.05** | **0.294** |
| **2002** | **1.1120** | **0.37** | **0.710** |
| **2003** | **0.7135** | **-1.23** | **0.217** |
| **2004** | **0.4568** | **-3.21** | **<0.01** |
| **2005** | **0.2774** | **-5.22** | **<0.01** |
| **2006** | **0.3708** | **-3.65** | **<0.01** |
| **2007** | **0.5720** | **-2.12** | **0.034** |
| **2008** | **0.5603** | **-2.27** | **0.023** |
| **2009** | **0.4560** | **-3.05** | **<0.01** |
| **2010** | **0.5490** | **-2.32** | **0.021** |
| **2011** | **0.6264** | **-1.25** | **0.213** |
| **2012** | **1.5820** | **1.09** | **0.277** |
| **2013** | **1.5609** | **0.88** | **0.381** |
| **2014** | **5.0287** | **2.09** | **0.037** |
| **2015** | **1.4338** | **0.78** | **0.436** |
| **2016** | **4.4551** | **2.15** | **0.032** |
| **2017** | **4.1429** | **2.52** | **0.012** |
| **2018** | **2.9014** | **2.07** | **0.038** |
| **2019** | **2.6807** | **1.82** | **0.068** |
| **Constant** | **3.8150** | **17.77** | **<0.01** |

**Wald Chi^2^ = 1172.33 (p<0.01); N = 37,238**

**
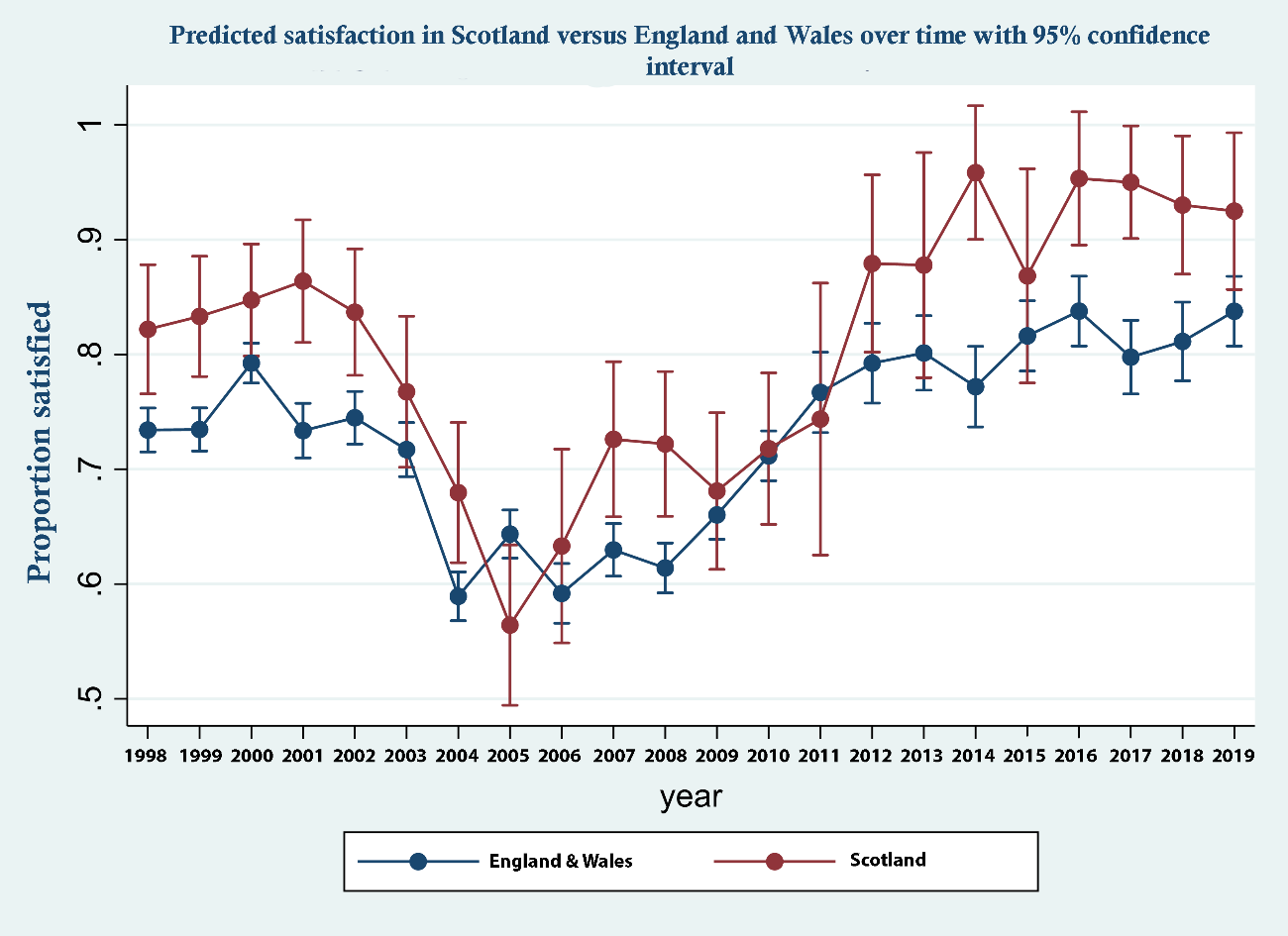
**

**Figure S2: Satisfaction with NHS dental services comparing Scotland with England and Wales over time.**

**Table S3 Predicted satisfaction with publicly funded dental services of who are over 65 versus who are 65 and under.**

| Independent variable | Odds Ratio | Std. Err. | z | P>\|z\| |
| --- | --- | --- | --- | --- |
|  |  |  |  |  |
|  |  |  |  |  |
| Over 65 | **1.4360** | **.1934** | **2.69** | **0.007** |
|  |  |  |  |  |
| Had a degree | **.7671** | **.0265** | **-7.66** | **0.000** |
|  |  |  |  |  |
| Had Dependent Child in Household | **1.0924** | **.0318** | **3.03** | **0.002** |
|  |  |  |  |  |
| Married | **.8590** | **.0258** | **-5.05** | **0.000** |
|  |  |  |  |  |
| Income  Quartile (relative to 1) |  |  |  |  |
| 2 | **.9310** | **.0363** | **-1.83** | **0.067** |
| 3 | **.8972** | **.0371** | **-2.62** | **0.009** |
| 4 | **.8257** | **.0364** | **-4.33** | **0.000** |
|  |  |  |  |  |
| Resides in Scotland | **1.4409** | **.0683** | **7.70** | **0.000** |
|  |  |  |  |  |
| White | **.8707** | **.0447** | **-2.69** | **0.007** |
|  |  |  |  |  |
| Male | **.9809** | **.0257** | **-0.73** | **0.465** |
|  |  |  |  |  |
| Year and Resident in England/Wales (relative to 1998 and living in Scotland) | | | | |
| 1999 | **1.0166** | **.0762** | **0.22** | **0.825** |
| 2000 | **1.3969** | **.1087** | **4.29** | **0.000** |
| 2001 | **1.0107** | **.0853** | **0.13** | **0.899** |
| 2002 | **1.0592** | **.0884** | **0.69** | **0.491** |
| 2003 | **.9633** | **.0794** | **-0.45** | **0.651** |
| 2004 | **.5294** | **.0378** | **-8.90** | **0.000** |
| 2005 | **.6120** | **.0445** | **-6.75** | **0.000** |
| 2006 | **.5108** | **.0407** | **-8.42** | **0.000** |
| 2007 | **.6258** | **.0473** | **-6.20** | **0.000** |
| 2008 | **.5875** | **.0430** | **-7.26** | **0.000** |
| 2009 | **.6970** | **.0516** | **-4.87** | **0.000** |
| 2010 | **.8839** | **.0691** | **-1.58** | **0.115** |
| 2011 | **1.0933** | **.1273** | **0.77** | **0.444** |
| 2012 | **1.4589** | **.1870** | **2.95** | **0.003** |
| 2013 | **1.4756** | **.1838** | **3.12** | **0.002** |
| 2014 | **1.3094** | **.1632** | **2.16** | **0.031** |
| 2015 | **1.6551** | **.2092** | **3.99** | **0.000** |
| 2016 | **2.0515** | **.2798** | **5.27** | **0.000** |
| 2017 | **1.5521** | **.1933** | **3.53** | **0.000** |
| 2018 | **1.5689** | **.2131** | **3.32** | **0.001** |
| 2019 | **1.8764** | **.2519** | **4.69** | **0.000** |
| Year and Resident in Scotland (relative to 1998 and living in England/Wales) | | | | |
| 1988 | **.9517** | **.1579** | **-0.30** | **0.766** |
| 2000 | **1.241** | **.2220** | **1.21** | **0.227** |
| 2001 | **1.0816** | **.2152** | **0.39** | **0.694** |
| 2002 | **1.1233** | **.2210** | **0.59** | **0.555** |
| 2003 | **.6080** | **.1092** | **-2.77** | **0.006** |
| 2004 | **.4244** | **.0671** | **-5.41** | **0.000** |
| 2005 | **.5792** | **.0942** | **-3.36** | **0.001** |
| 2006 | **.4899** | **.0850** | **-4.11** | **0.000** |
| 2007 | **.5263** | **.0877** | **-3.85** | **0.000** |
| 2008 | **.4940** | **.0794** | **-4.39** | **0.000** |
| 2009 | **.5853** | **.0979** | **-3.20** | **0.001** |
| 2010 | **.7304** | **.1254** | **-1.83** | **0.067** |
| 2011 | **1.5902** | **.4699** | **1.57** | **0.116** |
| 2012 | **1.0818** | **.2665** | **0.32** | **0.750** |
| 2013 | **1.4241** | **.3817** | **1.32** | **0.187** |
| 2014 | **1.2443** | **.3013** | **0.90** | **0.367** |
| 2015 | **1.3363** | **.3319** | **1.17** | **0.243** |
| 2016 | **1.5636** | **.4361** | **1.60** | **0.109** |
| 2017 | **1.2613** | **.3007** | **0.97** | **0.330** |
| 2018 | **1.8060** | **.4917** | **2.17** | **0.030** |
| 2019 | **2.1228** | **.5765** | **2.77** | **0.006** |
|  |  |  |  |  |
| Constant | **3.7909** | **.2929** | **17.25** | **0.000** |

**Wald chi2(52) = 1184.37 (p<0.01); N = 37,238**

**
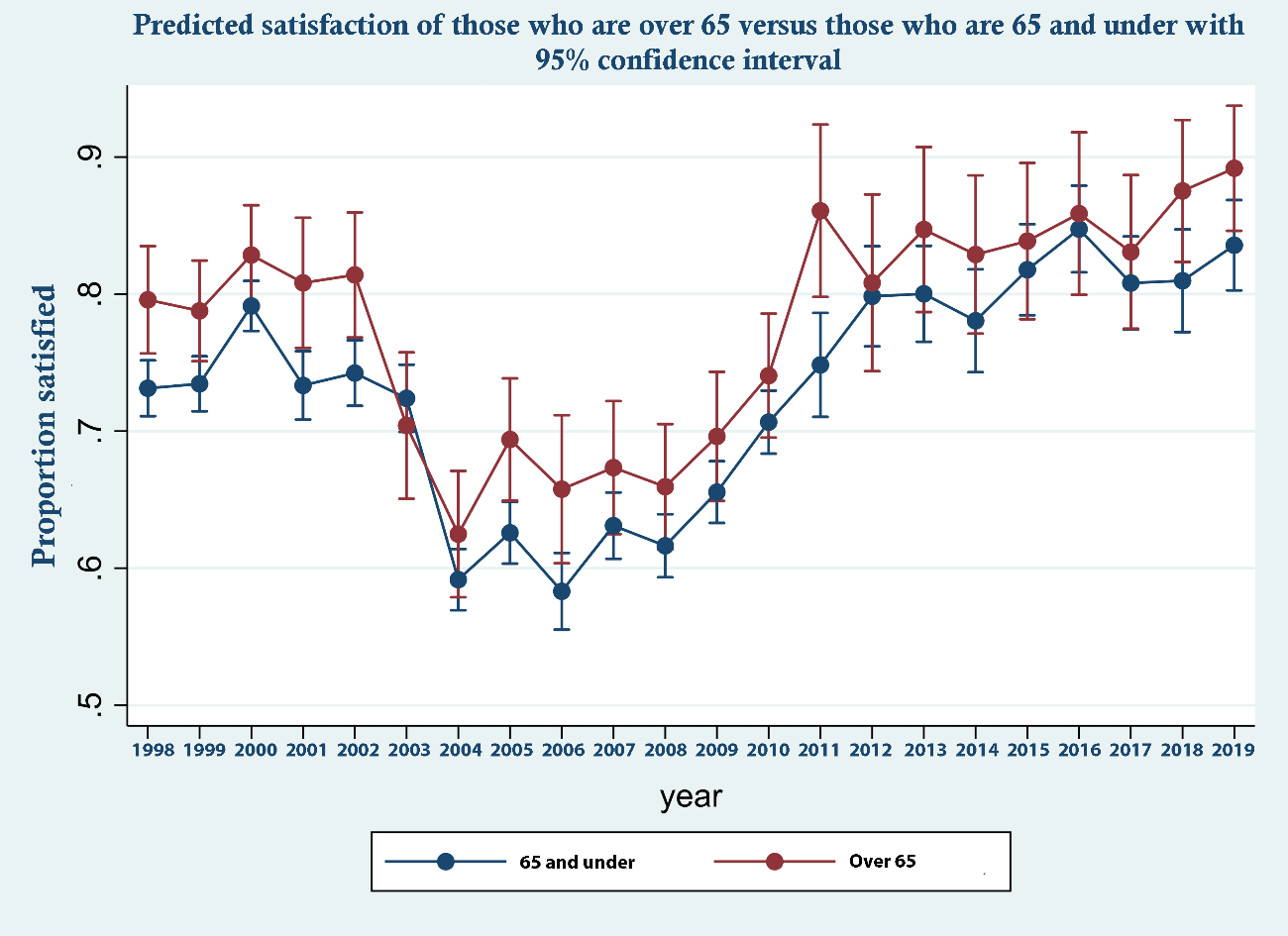
**

**Figure S3: Predicted satisfaction with publicly funded dental services of who are over 65 versus who are 65 and under.**

**TABLE S4 Predicted Satisfaction of Income Quartile Over Time**

| Independent variable | Odds Ratio | Std. Err. | z | P>\|z\| |
| --- | --- | --- | --- | --- |
|  |  |  |  |  |
|  |  |  |  |  |
| Over65 | **1.2582** | **.0471** | **6.13** | **0.000** |
|  |  |  |  |  |
| Had a degree | **.7632** | **.0264** | **-7.78** | **0.000** |
|  |  |  |  |  |
|  |  |  |  |  |
|  |  |  |  |  |
| Had Dependent Child in Household | **1.0893** | **.0318** | **2.93** | **0.003** |
|  |  |  |  |  |
| Married | **.8619** | **.0259** | **-4.94** | **0.000** |
|  |  |  |  |  |
| Income Quartile (relative to 1) |  |  |  |  |
| 2 | **.8070** | **.1158** | **-1.49** | **0.135** |
| 3 | **.6917** | **.0978** | **-2.61** | **0.009** |
| 4 | **.7085** | **.1039** | **-2.35** | **0.019** |
|  |  |  |  |  |
| Resides in Scotland | **1.4481** | **.0687** | **7.80** | **0.000** |
|  |  |  |  |  |
| White | **.8689** | **.0447** | **-2.73** | **0.006** |
|  |  |  |  |  |
| Male | **.9810** | **.0258** | **-0.73** | **0.468** |
|  |  |  |  |  |
| Year relative to income quartile |  |  |  |  |
| 1 1999 | **.95797** | **.1429** | **-0.29** | **0.773** |
| 1 2000 | **1.2989** | **.2120** | **1.60** | **0.109** |
| 1 2001 | **.9229** | **.1575** | **-0.47** | **0.639** |
| 1 2002 | **1.0729** | **.1852** | **0.41** | **0.683** |
| 1 2003 | **.7515** | **.1199** | **-1.79** | **0.074** |
| 1 2004 | **.4815** | **.0650** | **-5.41** | **0.000** |
| 1 2005 | **.6287** | **.0866** | **-3.37** | **0.001** |
| 1 2006 | **.5546** | **.0858** | **-3.81** | **0.000** |
| 1 2007 | **.4787** | **.0678** | **-5.20** | **0.000** |
| 1 2008 | **.5141** | **.0720** | **-4.75** | **0.000** |
| 1 2009 | **.5047** | **.0729** | **-4.73** | **0.000** |
| 1 2010 | **.6350** | **.0917** | **-3.14** | **0.002** |
| 1 2011 | **.6236** | **.1312** | **-2.24** | **0.025** |
| 1 2012 | **.9849** | **.2273** | **-0.07** | **0.948** |
| 1 2013 | **.8498** | **.1809** | **-0.76** | **0.445** |
| 1 2014 | **1.0647** | **.2402** | **0.28** | **0.781** |
| 1 2015 | **1.4207** | **.3071** | **1.62** | **0.104** |
| 1 2016 | **.9608** | **.2591** | **-0.15** | **0.882** |
| 1 2017 | **1.273** | **.3123** | **0.99** | **0.325** |
| 1 2018 | **.8624** | **.2087** | **-0.61** | **0.541** |
| 1 2019 | **1.4592** | **.3437** | **1.60** | **0.109** |
|  |  |  |  |  |
| 2 1999 | **.9971** | **.1321** | **-0.02** | **0.983** |
| 2 2000 | **1.5243** | **.2118** | **3.03** | **0.002** |
| 2 2001 | **1.0266** | **.1604** | **0.17** | **0.866** |
| 2 2002 | **1.0018** | **.1554** | **0.01** | **0.990** |
| 2 2003 | **.8868** | **.1325** | **-0.80** | **0.422** |
| 2 2004 | **.5196** | **.0675** | **-5.04** | **0.000** |
| 2 2005 | **.5119** | **.0675** | **-5.07** | **0.000** |
| 2 2006 | **.4558** | **.0629** | **-5.69** | **0.000** |
| 2 2007 | **.6554** | **.0952** | **-2.91** | **0.004** |
| 2 2008 | **.5498** | **.0719** | **-4.57** | **0.000** |
| 2 2009 | **.7279** | **.0950** | **-2.43** | **0.015** |
| 2 2010 | **.8932** | **.1303** | **-0.77** | **0.439** |
| 2 2011 | **1.230** | **.2800** | **0.91** | **0.363** |
| 2 2012 | **1.0914** | **.2560** | **0.37** | **0.709** |
| 2 2013 | **1.6367** | **.4211** | **1.91** | **0.056** |
| 2 2014 | **1.3842** | **.3181** | **1.41** | **0.157** |
| 2 2015 | **1.5551** | **.3921** | **1.75** | **0.080** |
| 2 2016 | **1.9295** | **.4498** | **2.82** | **0.005** |
| 2 2017 | **1.6413** | **.4080** | **1.99** | **0.046** |
| 2 2018 | **1.5942** | **.3689** | **2.02** | **0.044** |
| 2 2019 | **1.7966** | **.4091** | **2.57** | **0.010** |
|  |  |  |  |  |
| 3 1999 | **1.0029** | **.1306** | **0.02** | **0.982** |
| 3 2000 | **1.4923** | **.2015** | **2.96** | **0.003** |
| 3 2001 | **1.0646** | **.1556** | **0.43** | **0.669** |
| 3 2002 | **1.2307** | **.1770** | **1.44** | **0.149** |
| 3 2003 | **1.0020** | **.1384** | **0.01** | **0.988** |
| 3 2004 | **.4770** | **.0603** | **-5.85** | **0.000** |
| 3 2005 | **.6467** | **.0841** | **-3.35** | **0.001** |
| 3 2006 | **.6051** | **.0832** | **-3.65** | **0.000** |
| 3 2007 | **.6895** | **.0885** | **-2.89** | **0.004** |
| 3 2008 | **.7235** | **.0943** | **-2.48** | **0.013** |
| 3 2009 | **.8712** | **.1171** | **-1.03** | **0.305** |
| 3 2010 | **1.0319** | **.1434** | **0.23** | **0.821** |
| 3 2011 | **1.5706** | **.3440** | **2.06** | **0.039** |
| 3 2012 | **1.7043** | **.3822** | **2.38** | **0.017** |
| 3 2013 | **1.7001** | **.3757** | **2.40** | **0.016** |
| 3 2014 | **1.4800** | **.3371** | **1.72** | **0.085** |
| 3 2015 | **1.9082** | **.4358** | **2.83** | **0.005** |
| 3 2016 | **1.7769** | **.4081** | **2.50** | **0.012** |
| 3 2017 | **1.6838** | **.3718** | **2.36** | **0.018** |
| 3 2018 | **2.0534** | **.4969** | **2.97** | **0.003** |
| 3 2019 | **2.7051** | **.7004** | **3.84** | **0.000** |
|  |  |  |  |  |
| 4 1999 | **1.0655** | **.1455** | **0.46** | **0.642** |
| 4 2000 | **1.2278** | **.1718** | **1.47** | **0.143** |
| 4 2001 | **1.043** | **.1575** | **0.28** | **0.777** |
| 4 2002 | **.98433** | **.1470** | **-0.11** | **0.916** |
| 4 2003 | **.93102** | **.1423** | **-0.47** | **0.640** |
| 4 2004 | **.5559** | **.0726** | **-4.49** | **0.000** |
| 4 2005 | **.6265** | **.0826** | **-3.54** | **0.000** |
| 4 2006 | **.4393** | **.0668** | **-5.40** | **0.000** |
| 4 2007 | **.6174** | **.0852** | **-3.49** | **0.000** |
| 4 2008 | **.5141** | **.0682** | **-5.01** | **0.000** |
| 4 2009 | **.6284** | **.0838** | **-3.48** | **0.000** |
| 4 2010 | **.8882** | **.1247** | **-0.84** | **0.399** |
| 4 2011 | **1.2816** | **.2672** | **1.19** | **0.234** |
| 4 2012 | **1.7789** | **.4019** | **2.55** | **0.011** |
| 4 2013 | **1.7763** | **.3865** | **2.64** | **0.008** |
| 4 2014 | **1.2724** | **.2696** | **1.14** | **0.256** |
| 4 2015 | **1.5344** | **.3185** | **2.06** | **0.039** |
| 4 2016 | **3.3299** | **.8195** | **4.89** | **0.000** |
| 4 2017 | **1.4421** | **.2765** | **1.91** | **0.056** |
| 4 2018 | **2.278** | **.5671** | **3.31** | **0.001** |
| 4 2019 | **1.7600** | **.4118** | **2.42** | **0.016** |
| Constant | **4.4930** | **.5288** | **12.76** | **0.000** |

**Wald chi2(94) = 1259.75 (p<0.01); N = 37,238**

**
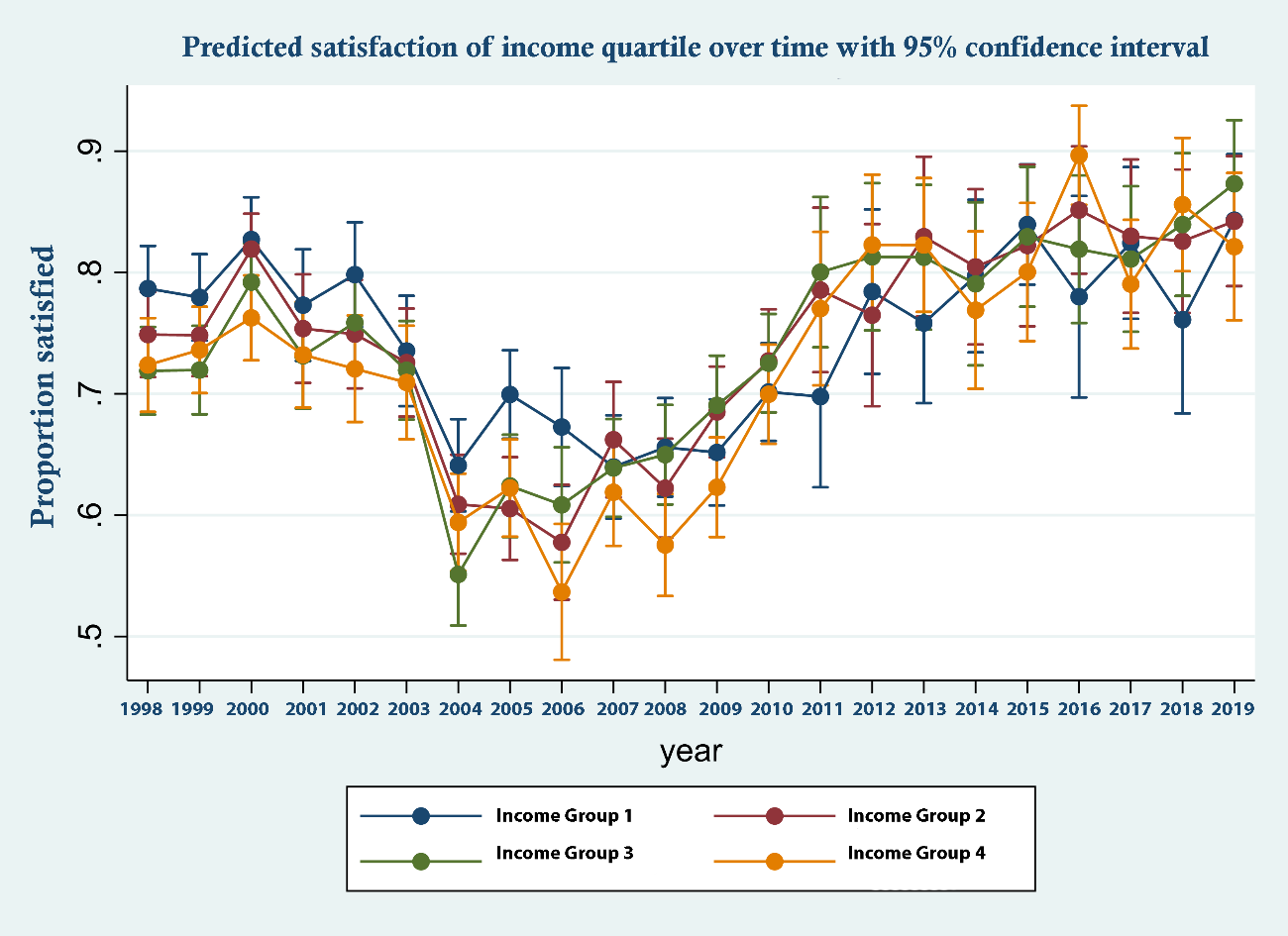
**

**Figure S4: Predicted Satisfaction of Income Quartile Over Time**

**TABLE S5 Predicted satisfaction with publicly funded dental services of household who have dependent child versus who don’t.**

| Independent variable | Odds Ratio | Std. Err | z | P>\|z\| |
| --- | --- | --- | --- | --- |
|  |  |  |  |  |
| Over65 |  |  |  |  |
| (yes=1) | **1.2813** | **.0479** | **6.62** | **0.000** |
|  |  |  |  |  |
| Had a degree | **.7665** | **.0265** | **-7.68** | **0.000** |
|  |  |  |  |  |
|  |  |  |  |  |
|  |  |  |  |  |
| Had Dependent Child in Household | **1.0714** | **.1133** | **0.65** | **0.514** |
|  |  |  |  |  |
| Married | **.8604** | **.0259** | **-4.99** | **0.000** |
| \| |  |  |  |  |
| Income  Quartile (relative to 1) |  |  |  |  |
| 2 | **.9313** | **.0361** | **-1.83** | **0.068** |
| 3 | **.8969** | **.0370** | **-2.63** | **0.009** |
| 4 | **.8272** | **.0364** | **-4.30** | **0.000** |
|  | | | |  |
| Resides in Scotland | **1.4388** | **.0683** | **7.67** | **0.000** |
|  |  |  |  |  |
| White | **.8709** | **.0447** | **-2.69** | **0.007** |
|  |  |  |  |  |
| Male | **.9808** | **.0258** | **-0.73** | **0.462** |
|  |  | | | |
| Year and Resident in England/Wales (relative to 1998 and living in Scotland) | | | | |
| 1999 | **1.0344** | **.0838** | **0.42** | **0.676** |
| 2000 | **1.3631** | **.1167** | **3.62** | **0.000** |
| 2001 | **.9876** | **.0934** | **-0.13** | **0.896** |
| 2002 | **1.1328** | **.1087** | **1.30** | **0.194** |
| 2003 | **.8375** | **.0760** | **-1.95** | **0.051** |
| 2004 | **.5025** | **.0394** | **-8.77** | **0.000** |
| 2005 | **.6030** | **.0480** | **-6.34** | **0.000** |
| 2006 | **.4941** | **.0428** | **-8.13** | **0.000** |
| 2007 | **.5967** | **.0501** | **-6.15** | **0.000** |
| 2008 | **.5800** | **.0478** | **-6.60** | **0.000** |
| 2009 | **.6195** | **.0511** | **-5.80** | **0.000** |
| 2010 | **.8916** | **.0793** | **-1.29** | **0.197** |
| 2011 | **1.1387** | **.1569** | **0.94** | **0.346** |
| 2012 | **1.5761** | **.2435** | **2.94** | **0.003** |
| 2013 | **1.5724** | **.2196** | **3.24** | **0.001** |
| 2014 | **1.3302** | **.1894** | **2.00** | **0.045** |
| 2015 | **1.6238** | **.2316** | **3.40** | **0.001** |
| 2016 | **2.1350** | **.3365** | **4.81** | **0.000** |
| 2017 | **1.4848** | **.2055** | **2.86** | **0.004** |
| 2018 | **1.5750** | **.2484** | **2.88** | **0.004** |
| 2019 | **2.0123** | **.3154** | **4.46** | **0.000** |
| Year and Resident in Scotland (relative to 1998 and living in England/Wales) | | | | |
| 1999 | **.9578** | **.1193** | **-0.35** | **0.730** |
| 2000 | **1.3935** | **.1790** | **2.58** | **0.010** |
| 2001 | **1.0858** | **.1464** | **0.61** | **0.542** |
| 2002 | **.9581** | **.1241** | **-0.33** | **0.742** |
| 2003 | **1.0357** | **.1386** | **0.26** | **0.793** |
| 2004 | **.5335** | **.0620** | **-5.41** | **0.000** |
| 2005 | **.6162** | **.0733** | **-4.07** | **0.000** |
| 2006 | **.5429** | **.0706** | **-4.69** | **0.000** |
| 2007 | **.6357** | **.0763** | **-3.77** | **0.000** |
| 2008 | **.5652** | **.0640** | **-5.03** | **0.000** |
| 2009 | **.8107** | **.0954** | **-1.78** | **0.075** |
| 2010 | **.8281** | **.0989** | **-1.58** | **0.114** |
| 2011 | **1.1301** | **.1975** | **0.70** | **0.484** |
| 2012 | **1.2360** | **.2172** | **1.21** | **0.228** |
| 2013 | **1.3377** | **.2528** | **1.54** | **0.124** |
| 2014 | **1.2688** | **.2290** | **1.32** | **0.187** |
| 2015 | **1.5667** | **.2909** | **2.42** | **0.016** |
| 2016 | **1.7638** | **.3463** | **2.89** | **0.004** |
| 2017 | **1.5337** | **.2830** | **2.32** | **0.020** |
| 2018 | **1.6615** | **.3224** | **2.62** | **0.009** |
| 2019 | **1.7855** | **.3468** | **2.98** | **0.003** |
|  |  |  |  |  |
| Constant | **3.8728** | **.3109** | **16.86** | **0.000** |

**Wald chi2(52) = 1179.79 (p<0.01); N = 37,238**

**
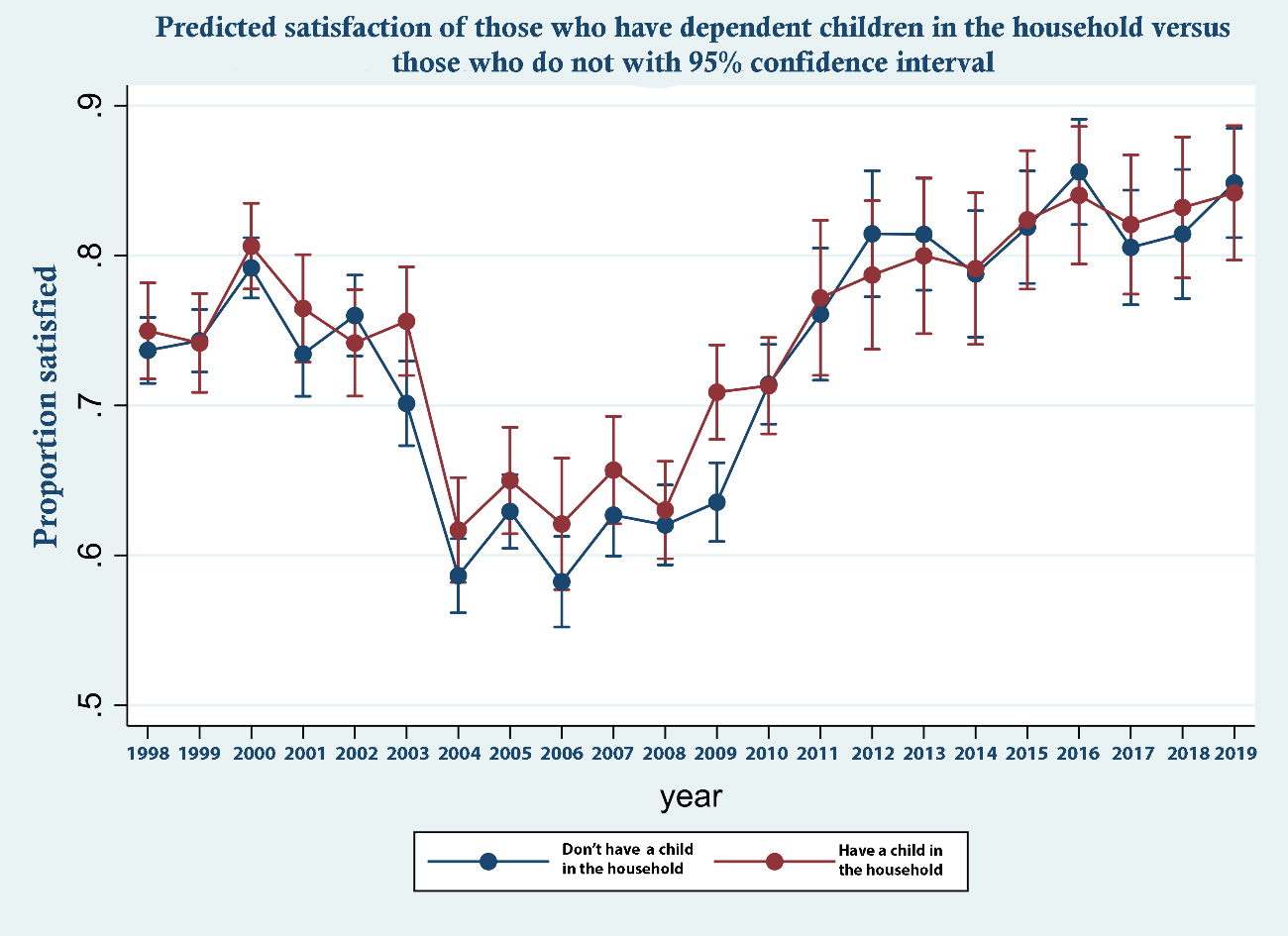
**

**Figure S5: Predicted satisfaction with publicly funded dental services of household who have dependent child versus who don’t.**

**TABLE S6 Predicted satisfaction with publicly funded dental services of people who are white versus who are not over time.**

| Independent Variable | Odds Ratio | Std. Err. | z | P>\|z\| |
| --- | --- | --- | --- | --- |
| Over 65 | **1.2789** | **.0478** | **6.57** | **0.000** |
|  |  |  |  |  |
| Had A Degree | **.7670** | **.0265** | **-7.66** | **0.000** |
|  |  |  |  |  |
| Had Dependent Child in Household | **1.0940** | **.0318** | **3.08** | **0.002** |
|  |  |  |  |  |
| Married | **.8587** | **.0258** | **-5.05** | **0.000** |
|  | | | | |
| Income quartile (relative to 1) |  | | | |
| 2 | **.9298** | **.0362** | **-1.87** | **0.062** |
| 3 | **.8957** | **.0370** | **-2.66** | **0.008** |
| 4 | **.8236** | **.0363** | **-4.40** | **0.000** |
|  | | | | |
| Resides in Scotland | **1.4412** | **.0684** | **7.69** | **0.000** |
|  |  |  |  |  |
| White | **.7825** | **.1678** | **-1.14** | **0.253** |
|  |  |  |  |  |
| Male | **.9817** | **.0258** | **-0.70** | **0.485** |
|  | | | | |
| Year and Resident in England/Wales (relative to 1998 and living in Scotland) | | | | |
| 1999 | **1.0230** | **.3392** | **0.07** | **0.945** |
| 2000 | **1.0973** | **.3328** | **0.31** | **0.759** |
| 2001 | **.6715** | **.2161** | **-1.24** | **0.216** |
| 2002 | **.5557** | **.1587** | **-2.06** | **0.040** |
| 2003 | **.7032** | **.2015** | **-1.23** | **0.219** |
| 2004 | **.6540** | **.1860** | **-1.49** | **0.136** |
| 2005 | **.7426** | **.2038** | **-1.08** | **0.278** |
| 2006 | **.5600** | **.1565** | **-2.07** | **0.038** |
| 2007 | **.6820** | **.1831** | **-1.42** | **0.154** |
| 2008 | **.5372** | **.1396** | **-2.39** | **0.017** |
| 2009 | **.6452** | **.1726** | **-1.64** | **0.101** |
| 2010 | **.6194** | **.1717** | **-1.73** | **0.084** |
| 2011 | **1.1614** | **.4613** | **0.38** | **0.706** |
| 2012 | **.8807** | **.3617** | **-0.31** | **0.757** |
| 2013 | **1.2828** | **.4551** | **0.70** | **0.483** |
| 2014 | **1.2432** | **.4785** | **0.57** | **0.572** |
| 2015 | **1.2078** | **.4276** | **0.53** | **0.594** |
| 2016 | **1.1717** | **.4799** | **0.39** | **0.699** |
| 2017 | **1.1414** | **.4332** | **0.35** | **0.728** |
| 2018 | **1.5722** | **.6148** | **1.16** | **0.247** |
| 2019 | **1.2981** | **.5128** | **0.66** | **0.509** |
|  | | | | |
| Year and Resident in Scotland (relative to 1998 and living in England/Wales) | | | | |
| 1999 | **1.0075** | **.0703** | **0.11** | **0.914** |
| 2000 | **1.3964** | **.1024** | **4.55** | **0.000** |
| 2001 | **1.0503** | **.0838** | **0.62** | **0.538** |
| 2002 | **1.1299** | **.0905** | **1.52** | **0.127** |
| 2003 | **.9209** | **.0718** | **-1.06** | **0.291** |
| 2004 | **.5050** | **.0338** | **-10.19** | **0.000** |
| 2005 | **.5946** | **.0406** | **-7.60** | **0.000** |
| 2006 | **.5014** | **.0375** | **-9.21** | **0.000** |
| 2007 | **.5981** | **.0426** | **-7.20** | **0.000** |
| 2008 | **.5741** | **.0396** | **-8.03** | **0.000** |
| 2009 | **.6807** | **.0476** | **-5.49** | **0.000** |
| 2010 | **.8869** | **.0655** | **-1.62** | **0.105** |
| 2011 | **1.1265** | **.1269** | **1.06** | **0.290** |
| 2012 | **1.4747** | **.1757** | **3.26** | **0.001** |
| 2013 | **1.4868** | **.1786** | **3.30** | **0.001** |
| 2014 | **1.3010** | **.1523** | **2.25** | **0.025** |
| 2015 | **1.6504** | **.1983** | **4.17** | **0.000** |
| 2016 | **2.0736** | **.2679** | **5.64** | **0.000** |
| 2017 | **1.5507** | **.1792** | **3.80** | **0.000** |
| 2018 | **1.6012** | **.2065** | **3.65** | **0.000** |
| 2019 | **1.9849** | **.2552** | **5.33** | **0.000** |
|  |  |  |  |  |
| Constant | **4.2641** | **.8954** | **6.91** | **0.000** |

**Wald chi2(52)= 1203.67 (p<0.01); N = 37,238**

**
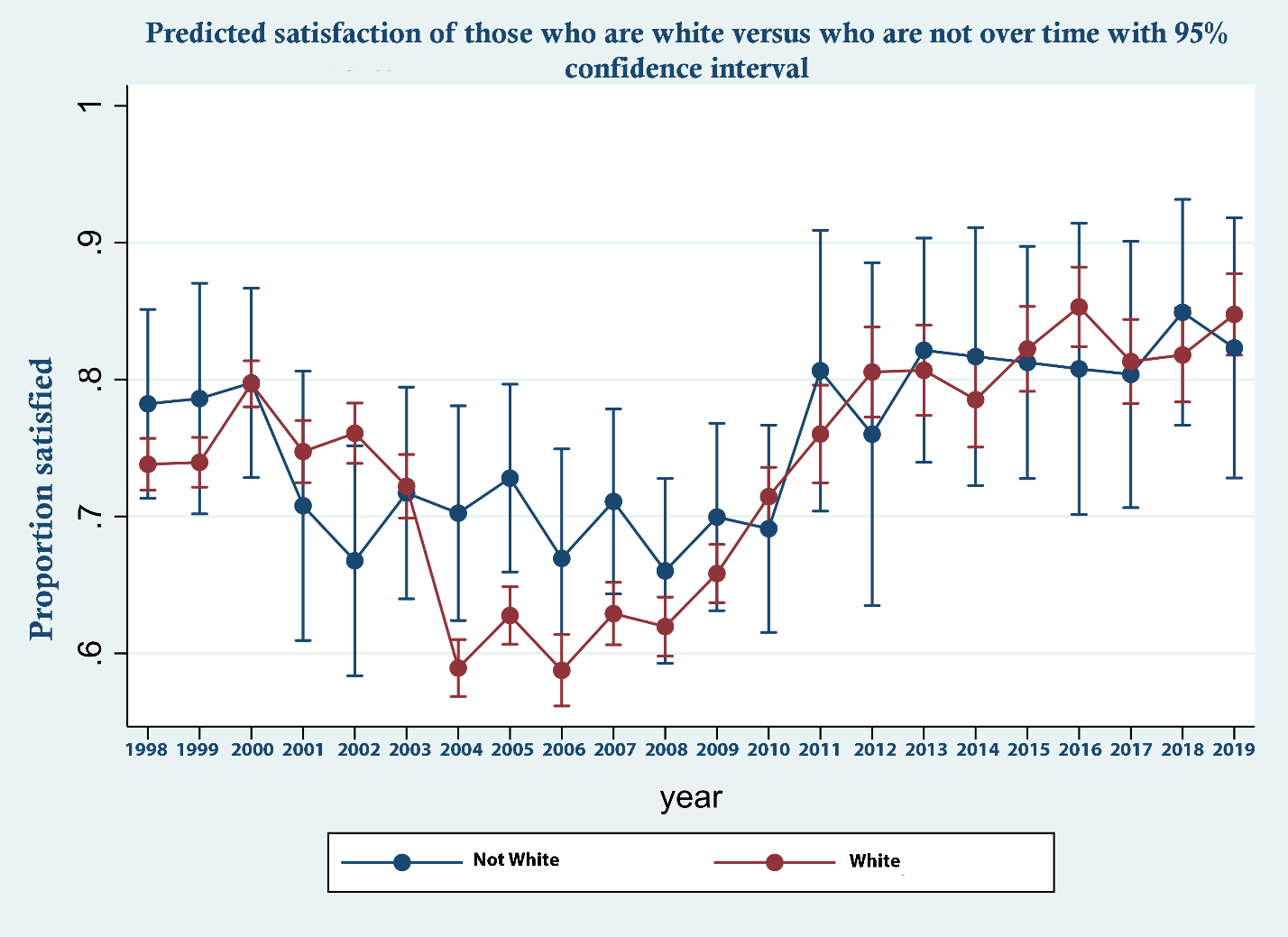
**

**Figure S6 : Predicted Satisfaction with Publicly Funded Dental Services of People Who are White Versus Who are not Over Time.**

**Table S7 Predicted satisfaction with publicly funded dental services of people who have a degree versus who do not.**

| Independent Variable | Odds Ratio | | | Std. Err. | | z | P>\|z\| |
| --- | --- | --- | --- | --- | --- | --- | --- |
|  |  | | |  | |  |  |
| Over65 | **1.2778** | | | **.04774** | | **6.56** | **0.000** |
|  |  | | |  | |  |  |
| Had A Degree | **.7027** | | | **.10816** | | **-2.29** | **0.022** |
|  |  | | |  | |  |  |
| Had Dependent Child in Household | **1.0925** | | | **.0318** | | **3.03** | **0.002** |
|  |  | | |  | |  |  |
| Married | **.8584** | | | **.0258** | | **-5.07** | **0.000** |
|  |  | | |  | |  |  |
| Income quartile (relative to 1) |  | | |  | |  |  |
| 2 | **.9293** | | | **.0361** | | **-1.88** | **0.060** |
| 3 | **.8972** | | | **.0371** | | **-2.62** | **0.009** |
| 4 | **.8268** | | | **.0364** | | **-4.31** | **0.000** |
|  |  | | |  | |  |  |
| Resides in Scotland | **1.4404** | | | **.0683** | | **7.69** | **0.000** |
|  |  | | |  | |  |  |
| Whit | **.8704** | | | **.0448** | | **-2.69** | **0.007** |
|  |  | | |  | |  |  |
| Male | **.9812** | | | **.0258** | | **-0.72** | **0.472** |
|  |  | | |  | |  |  |
| Year and Resident in England/Wales (relative to 1998 and living in Scotland) | | | | | | | |
| 1999 | **1.0292** | | | **.0757** | | **0.39** | **0.695** |
| 2000 | **1.3834** | | | **.1071** | | **4.19** | **0.000** |
| 2001 | **1.0125** | | | **.0854** | | **0.15** | **0.883** |
| 2002 | **1.0354** | | | **.0865** | | **0.42** | **0.677** |
| 2003 | **.8629** | | | **.0699** | | **-1.82** | **0.069** |
| 2004 | **.4991** | | | **.0351** | | **-9.86** | **0.000** |
| 2005 | **.6192** | | | **.0444** | | **-6.67** | **0.000** |
| 2006 | **.5003** | | | **.0392** | | **-8.82** | **0.000** |
| 2007 | **.6091** | | | **.0463** | | **-6.52** | **0.000** |
| 2008 | **.5880** | | | **.0429** | | **-7.27** | **0.000** |
| 2009 | **.6689** | | | **.0499** | | **-5.39** | **0.000** |
| 2010 | **.8272** | | | **.0655** | | **-2.39** | **0.017** |
| 2011 | **1.0924** | | | **.1322** | | **0.73** | **0.465** |
| 2012 | **1.2623** | | | **.1643** | | **1.79** | **0.074** |
| 2013 | **1.4128** | | | **.1864** | | **2.62** | **0.009** |
| 2014 | **1.2987** | | | **.1672** | | **2.03** | **0.042** |
| 2015 | **1.6441** | | | **.2187** | | **3.74** | **0.000** |
| 2016 | **1.7849** | | | **.2559** | | **4.04** | **0.000** |
| 2017 | **1.5534** | | | **.2090** | | **3.27** | **0.001** |
| 2018 | **1.3731** | | | **.1901** | | **2.29** | **0.022** |
| 2019 | **1.9757** | | | **.2827** | | **4.76** | **0.000** |
| Year and Resident in Scotland (relative to 1998 and living in England/Wales) | | | | | | | |
| 1999 | **.9038** | | | **.1729** | | **-0.53** | **0.598** |
| 2000 | **1.3714** | | | **.2628** | | **1.65** | **0.099** |
| 2001 | **1.0868** | | | **.2200** | | **0.41** | **0.681** |
| 2002 | **1.2372** | | | **.2489** | | **1.06** | **0.290** |
| 2003 | **1.1358** | | | **.2293** | | **0.63** | **0.528** |
| 2004 | **.5971** | | | **.1053** | | **-2.92** | **0.003** |
| 2005 | **.5804** | | | **.1047** | | **-3.02** | **0.003** |
| 2006 | **.5662** | | | **.1077** | | **-2.99** | **0.003** |
| 2007 | **.6435** | | | **.1132** | | **-2.51** | **0.012** |
| 2008 | **.5473** | | | **.0956** | | **-3.45** | **0.001** |
| 2009 | **.7491** | | | **.1297** | | **-1.67** | **0.095** |
| 2010 | **1.0043** | | | **.1778** | | **0.02** | **0.980** |
| 2011 | **1.3337** | | | **.3323** | | **1.16** | **0.248** |
| 2012 | **1.9597** | | | **.4930** | | **2.67** | **0.007** |
| 2013 | **1.6912** | | | **.3995** | | **2.22** | **0.026** |
| 2014 | **1.3795** | | | **.3311** | | **1.34** | **0.180** |
| 2015 | **1.5937** | | | **.3740** | | **1.99** | **0.047** |
| 2016 | **2.5922** | | | **.6487** | | **3.81** | **0.000** |
| 2017 | **1.4874** | | | **.3291** | | **1.79** | **0.073** |
| 2018 | | **2.4882** | **.6561** | | **3.46** | | **0.001** |
| 2019 | | **1.8842** | **.4687** | | **2.55** | | **0.011** |
|  | |  |  | |  | |  |
| Constant | | **3.8952** | **.2982** | | **17.76** | | **0.000** |

**Wald chi2(52) = 1185.82** **(p<0.01); N = 37,238**

**
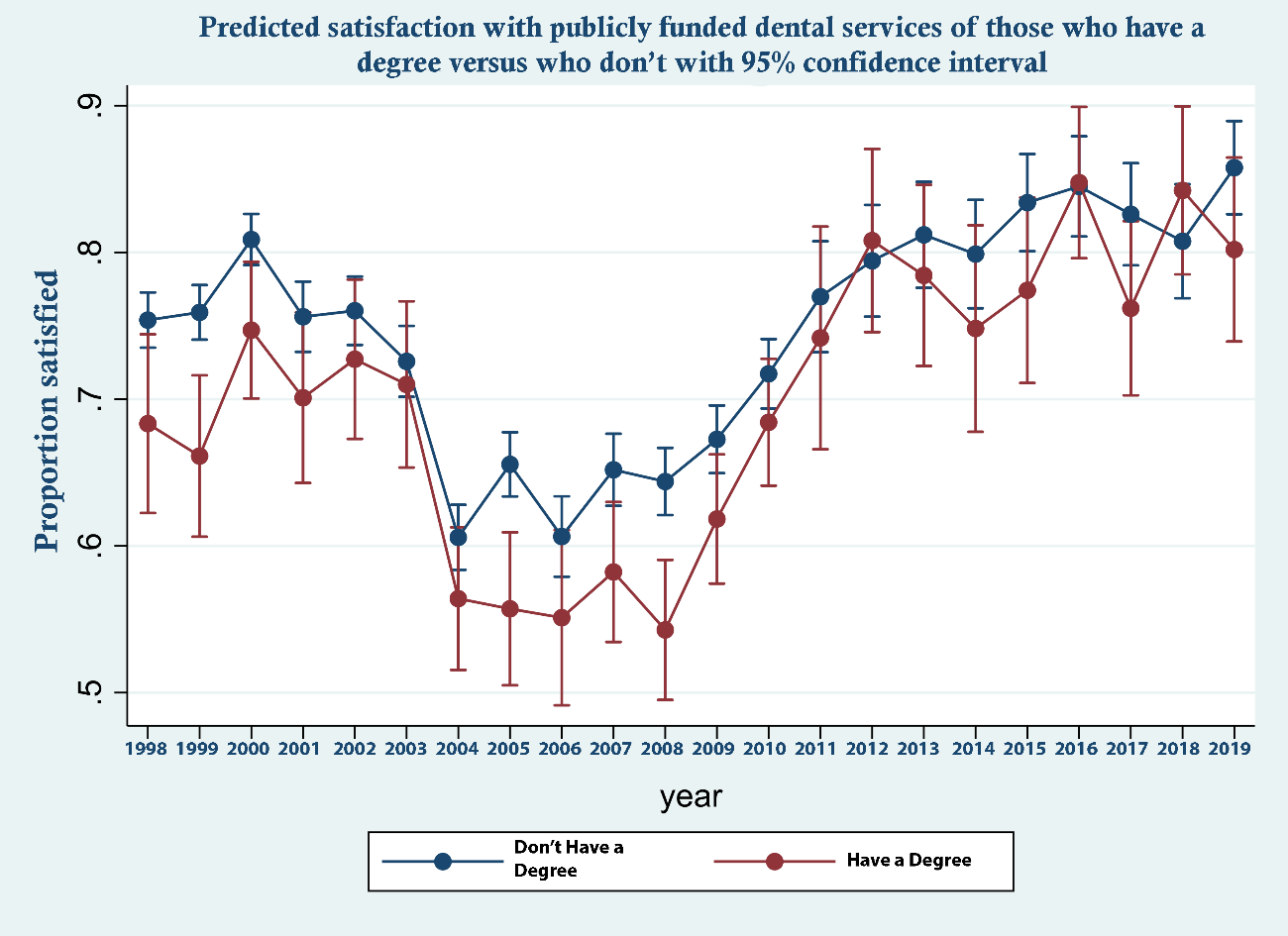
**

**Figure S7: Predicted satisfaction with publicly funded dental services of people who have a degree versus who do not.**

**Table S8 Predicted satisfaction with publicly funded dental services of male versus female.**

| Independent Variable | Odds Ratio | Std. Err. | z | P>\|z\| |
| --- | --- | --- | --- | --- |
|  |  |  |  |  |
| Over65 | **1.2809** | **.04791** | **6.62** | **0.000** |
|  |  |  |  |  |
| Had A Degree | **.7668** | **.0265** | **-7.67** | **0.000** |
|  |  |  |  |  |
| Had Dependent Child in Household | **1.0939** | **.0319** | **3.08** | **0.002** |
|  |  |  |  |  |
| Married | **.8580** | **.0258** | **-5.09** | **0.000** |
|  |  |  |  |  |
| Income quartile (relative to 1) |  |  |  |  |
| 2 | **.9305** | **.0362** | **-1.85** | **0.064** |
| 3 | **.8983** | **.0371** | **-2.59** | **0.010** |
| 4 | **.8275** | **.0364** | **-4.29** | **0.000** |
|  |  |  |  |  |
| Resides in Scotland | **1.4402** | **.0684** | **7.68** | **0.000** |
|  |  |  |  |  |
| White | **.8708** | **.0447** | **-2.69** | **0.007** |
|  |  |  |  |  |
| Male | **.8423** | **.0826** | **-1.75** | **0.080** |
|  |  | |  |  |
| Year and Resident in England/Wales (relative to 1998 and living in Scotland) | | | | |
| 1999 | **1.0018** | **.0944** | **0.02** | **0.985** |
| 2000 | **1.3434** | **.1317** | **3.01** | **0.003** |
| 2001 | **.8868** | **.0941** | **-1.13** | **0.258** |
| 2002 | **.9752** | **.1028** | **-0.24** | **0.812** |
| 2003 | **.8571** | **.0877** | **-1.51** | **0.132** |
| 2004 | **.4956** | **.0440** | **-7.90** | **0.000** |
| 2005 | **.5746** | **.0517** | **-6.15** | **0.000** |
| 2006 | **.4639** | **.0457** | **-7.79** | **0.000** |
| 2007 | **.5912** | **.0552** | **-5.63** | **0.000** |
| 2008 | **.5024** | **.0447** | **-7.73** | **0.000** |
| 2009 | **.6595** | **.0602** | **-4.56** | **0.000** |
| 2010 | **.6961** | **.0660** | **-3.81** | **0.000** |
| 2011 | **1.1259** | **.1633** | **0.82** | **0.413** |
| 2012 | **1.2840** | **.1947** | **1.65** | **0.099** |
| 2013 | **1.1708** | **.1715** | **1.08** | **0.281** |
| 2014 | **1.3001** | **.2012** | **1.70** | **0.090** |
| 2015 | **1.2102** | **.1750** | **1.32** | **0.187** |
| 2016 | **1.4830** | **.2294** | **2.55** | **0.011** |
| 2017 | **1.5947** | **.2465** | **3.02** | **0.003** |
| 2018 | **1.6267** | **.2644** | **2.99** | **0.003** |
| 2019 | **1.5491** | **.2442** | **2.78** | **0.005** |
| Year and Resident in Scotland (relative to 1998 and living in England/Wales) | | | | |
| 1999 | **1.0198** | **.1016** | **0.20** | **0.843** |
| 2000 | **1.4106** | **.1476** | **3.29** | **0.001** |
| 2001 | **1.1923** | **.1353** | **1.55** | **0.121** |
| 2002 | **1.1719** | **.1320** | **1.41** | **0.159** |
| 2003 | **.9532** | **.1055** | **-0.43** | **0.666** |
| 2004 | **.5313** | **.0510** | **-6.57** | **0.000** |
| 2005 | **.6480** | **.0635** | **-4.43** | **0.000** |
| 2006 | **.5642** | **.0599** | **-5.38** | **0.000** |
| 2007 | **.6339** | **.0646** | **-4.47** | **0.000** |
| 2008 | **.6638** | **.0665** | **-4.09** | **0.000** |
| 2009 | **.7053** | **.0709** | **-3.47** | **0.001** |
| 2010 | **1.0907** | **.1175** | **0.81** | **0.420** |
| 2011 | **1.1524** | **.1846** | **0.89** | **0.376** |
| 2012 | **1.5344** | **.2638** | **2.49** | **0.013** |
| 2013 | **1.8787** | **.3308** | **3.58** | **0.000** |
| 2014 | **1.3240** | **.2120** | **1.75** | **0.080** |
| 2015 | **2.1944** | **.3942** | **4.37** | **0.000** |
| 2016 | **2.8682** | **.5865** | **5.15** | **0.000** |
| 2017 | **1.4418** | **.2288** | **2.31** | **0.021** |
| 2018 | **1.6193** | **.2899** | **2.69** | **0.007** |
| 2019 | **2.3813** | **.4499** | **4.59** | **0.000** |
|  |  |  |  |  |
| Constant | **4.1375** | **.3591** | **16.36** | **0.000** |

**Wald chi2(52) = 1195.79 (p<0.01); N = 37,238**

**
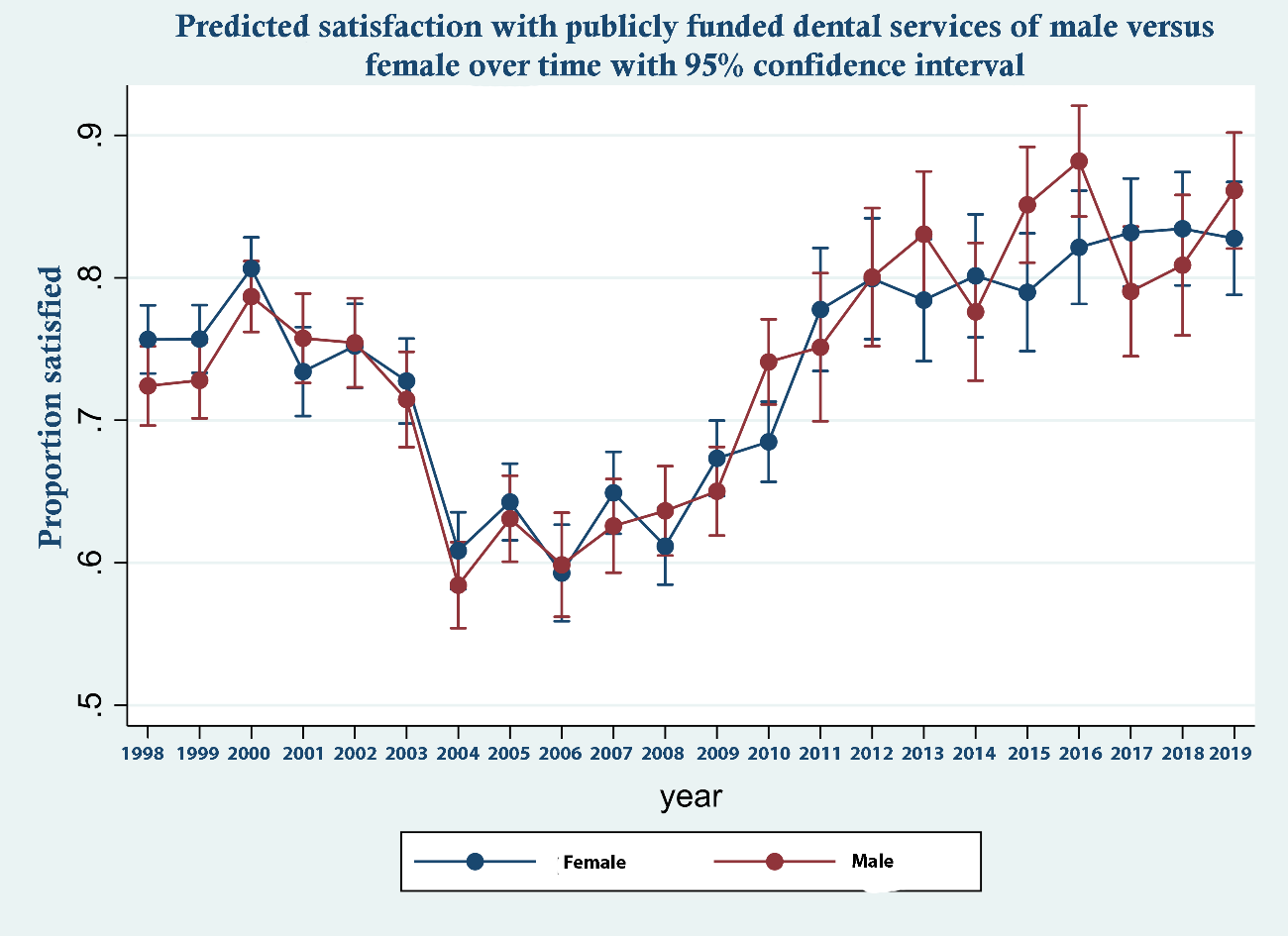
**

**Figure S8 : Predicted satisfaction with publicly funded dental services of male versus female.**

**Table S9 Predicted satisfaction with publicly funded dental services of married versus who are not.**

| Independent Variable | Odds Ratio | Std. Err. | | z | P>\|z\| |
| --- | --- | --- | --- | --- | --- |
|  |  |  |  | |  |
| Over65 | **1.2765** | **.04779** | **6.52** | | **0.000** |
|  |  |  |  | |  |
| Had Degree | **.7649** | **.02647** | **-7.74** | | **0.000** |
|  |  |  |  | |  |
| Had Dependent Child In Household | **1.0907** | **.0318** | **2.98** | | **0.003** |
|  |  |  |  | |  |
| Married | **.8830** | **.0913** | **-1.20** | | **0.229** |
|  |  |  |  | |  |
| Incom2002e Quartile (Relative To 1) |  |  |  | |  |
| 2 | **.9306** | **.0362** | **-1.85** | | **0.065** |
| 3 | **.8977** | **.0371** | **-2.61** | | **0.009** |
| 4 | **.8273** | **.0364** | **-4.30** | | **0.000** |
|  |  |  |  | |  |
| Resides In Scotland | **1.4397** | **.0683** | **7.68** | | **0.000** |
|  |  |  |  | |  |
| White | **.8707** | **.0448** | **-2.69** | | **0.007** |
|  |  |  |  | |  |
| Male | **.9825** | **.0258** | **-0.67** | | **0.504** |
|  |  |  |  | |  |
| Year And Resident in England/Wales (Relative To 1998 And Living In Scotland) | | | | | |
| 1999 | **1.0646** | **.1220** | **0.55** | | **0.584** |
| 2000 | **1.2244** | **.1478** | **1.68** | | **0.093** |
| 2001 | **1.0997** | **.1450** | **0.72** | | **0.471** |
| 2002 | **1.1870** | **.1567** | **1.30** | | **0.194** |
| 2003 | **.9706** | **.1186** | **-0.24** | | **0.807** |
| 2004 | **.5679** | **.0620** | **-5.18** | | **0.000** |
| 2005 | **.6377** | **.0722** | **-3.97** | | **0.000** |
| 2006 | **.5362** | **.0659** | **-5.07** | | **0.000** |
| 2007 | **.6026** | **.0691** | **-4.41** | | **0.000** |
| 2008 | **.6568** | **.0735** | **-3.75** | | **0.000** |
| 2009 | **.5858** | **.0664** | **-4.71** | | **0.000** |
| 2010 | **.8453** | **.1002** | **-1.42** | | **0.157** |
| 2011 | **.9766** | **.1736** | **-0.13** | | **0.894** |
| 2012 | **1.4687** | **.2961** | **1.91** | | **0.057** |
| 2013 | **1.5791** | **.2861** | **2.52** | | **0.012** |
| 2014 | **1.4375** | **.2880** | **1.81** | | **0.070** |
| 2015 | **1.4742** | **.2736** | **2.09** | | **0.037** |
| 2016 | **1.7524** | **.3696** | **2.66** | | **0.008** |
| 2017 | **1.9964** | **.4256** | **3.24** | | **0.001** |
| 2018 | **1.6981** | **.3484** | **2.58** | | **0.010** |
| 2019 | **1.4228** | **.2702** | **1.86** | | **0.063** |
| Year And Resident in Scotland (Relative To 1998 And Living In England/Wales) | | | | | |
| 1999 | **.9839** | **.0829** | **-0.19** | | **0.848** |
| 2000 | **1.4486** | **.1276** | **4.21** | | **0.000** |
| 2001 | **.9838** | **.0941** | **-0.17** | | **0.865** |
| 2002 | **1.0133** | **.0956** | **0.14** | | **0.889** |
| 2003 | **.8713** | **.0818** | **-1.47** | | **0.143** |
| 2004 | **.4899** | **.0394** | **-8.85** | | **0.000** |
| 2005 | **.5948** | **.0485** | **-6.37** | | **0.000** |
| 2006 | **.4979** | **.0442** | **-7.85** | | **0.000** |
| 2007 | **.6164** | **.0529** | **-5.63** | | **0.000** |
| 2008 | **.5353** | **.0443** | **-7.55** | | **0.000** |
| 2009 | **.7338** | **.0615** | **-3.69** | | **0.000** |
| 2010 | **.8695** | **.0772** | **-1.57** | | **0.115** |
| 2011 | **1.2234** | **.1665** | **1.48** | | **0.138** |
| 2012 | **1.3699** | **.1923** | **2.24** | | **0.025** |
| 2013 | **1.4222** | **.2026** | **2.47** | | **0.013** |
| 2014 | **1.2437** | **.1679** | **1.62** | | **0.106** |
| 2015 | **1.6835** | **.2386** | **3.68** | | **0.000** |
| 2016 | **2.0625** | **.3100** | **4.82** | | **0.000** |
| 2017 | **1.3372** | **.1751** | **2.22** | | **0.027** |
| 2018 | **1.5725** | **.2372** | **3.00** | | **0.003** |
| 2019 | **2.2408** | **.3558** | **5.08** | | **0.000** |
|  |  |  |  | |  |
| Constant | **3.7800** | **.3721** | **13.51** | | **0.000** |

**Wald chi2(52) = 1189.05 (p<0.01); N = 37,238**

**
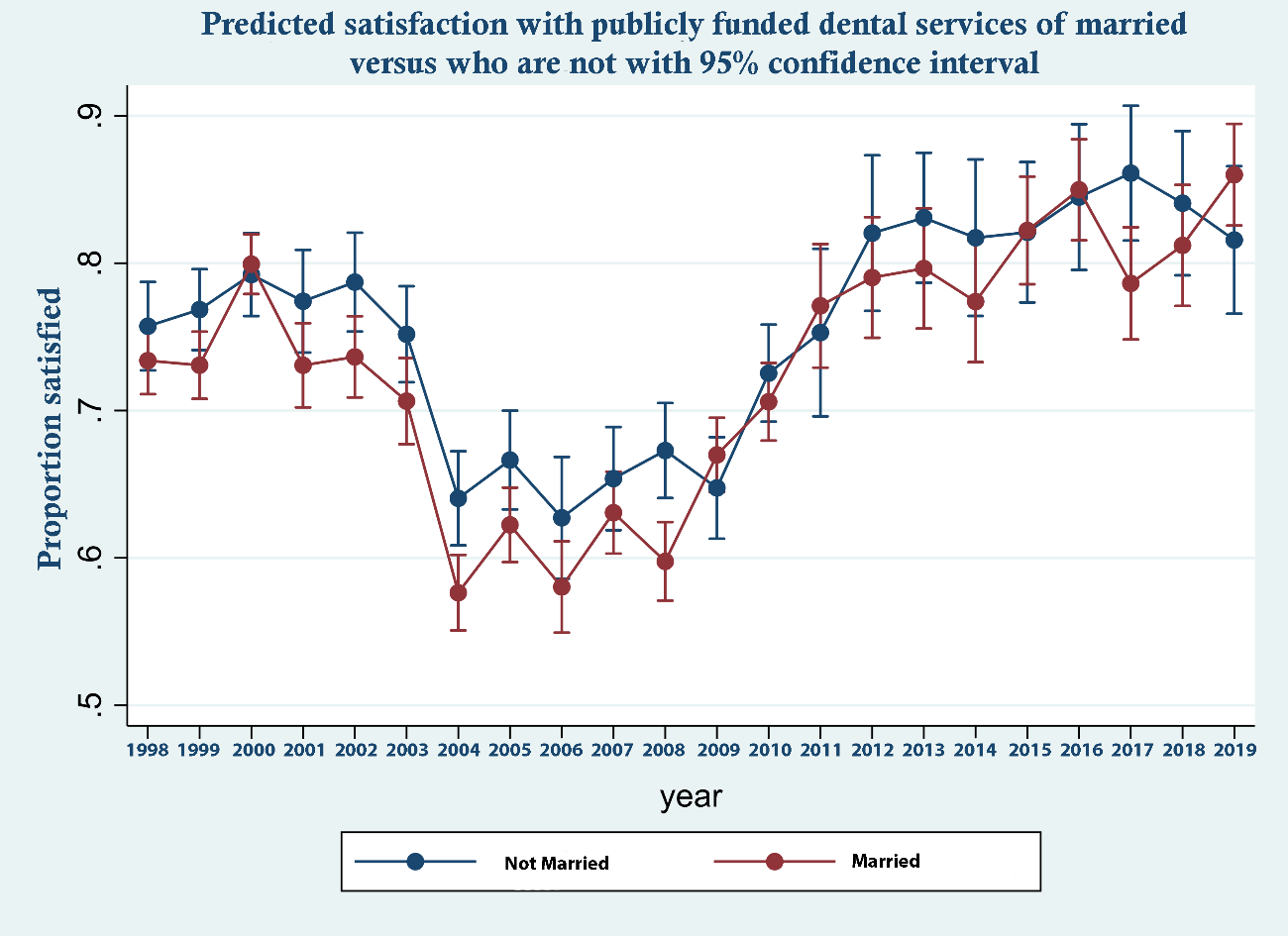
**

**Figure S9: Predicted satisfaction with publicly funded dental services of married versus who are not.**
