# Supplementary material for: Satisfaction with dental care services in Great Britain 1998–2019
Source: BMC Oral Health. 2022 Jul 26;22:308. doi: 10.1186/s12903-022-02343-7 (PMC9315088; doi:10.1186/s12903-022-02343-7)
Supplement: Supplementary file 3 — Additional file 3. Unweighted analysis. [file 12903_2022_2343_MOESM3_ESM.docx]

**Online Supplement 3**

**Table F1: Satisfaction with publicly funded dental services as a function of the variables shown**

**( unweighted)**

| Independent | Odds ratio | Std Error. | Z | P>\|z\| |
| --- | --- | --- | --- | --- |
| Over65 | **1.3486** | **.0460** | **8.75** | **0.000** |
|  |  |  |  |  |
| Had a degree | **.7629** | **.0241** | **-8.54** | **0.000** |
|  |  |  |  |  |
| Married | **.9066** | **.0245** | **-3.62** | **0.000** |
|  |  |  |  |  |
| Income Quartile (relative to 1) |  |  |  |  |
| 2 | **.9055** | **.0314** | **-2.86** | **0.004** |
| 3 | **.8665** | **.0322** | **-3.85** | **0.000** |
| 4 | **.7961** | **.0318** | **-5.70** | **0.000** |
|  |  |  |  |  |
| Male | **.9606** | **.0231** | **-1.67** | **0.095** |
|  |  |  |  |  |
| Resides in Scotland | **1.4682** | **.0639** | **8.82** | **0.000** |
|  |  |  |  |  |
| White | **.9017** | **.0412** | **-2.26** | **0.024** |
|  |  |  |  |  |
| Had Dependent Child in Household | **1.1395** | **.0309** | **4.81** | **0.000** |
|  |  |  |  |  |
| Year and Resident in England/Wales (relative to 1998 and living in Scotland) | | | | |
| 1999 | **.9875** | **.0623** | **-0.20** | **0.843** |
| 2000 | **1.4003** | **.0916** | **5.14** | **0.000** |
| 2001 | **1.0249** | **.0727** | **0.35** | **0.728** |
| 2002 | **1.0415** | **.0737** | **0.58** | **0.565** |
| 2003 | **.8646** | **.0602** | **-2.09** | **0.037** |
| 2004 | **.5039** | **.0303** | **-11.40** | **0.000** |
| 2005 | **.5776** | **.0353** | **-8.98** | **0.000** |
| 2006 | **.4739** | **.0315** | **-11.20** | **0.000** |
| 2007 | **.5850** | **.0370** | **-8.47** | **0.000** |
| 2008 | **.5403** | **.0332** | **-10.01** | **0.000** |
| 2009 | **.6621** | **.0410** | **-6.65** | **0.000** |
| 2010 | **.8263** | **.0538** | **-2.93** | **0.003** |
| 2011 | **1.0975** | **.1087** | **0.94** | **0.348** |
| 2012 | **1.3343** | **.1372** | **2.80** | **0.005** |
| 2013 | **1.3674** | **.1413** | **3.03** | **0.002** |
| 2014 | **1.3058** | **.1320** | **2.64** | **0.008** |
| 2015 | **1.5183** | **.1545** | **4.10** | **0.000** |
| 2016 | **1.7884** | **.2021** | **5.14** | **0.000** |
| 207 | **1.4737** | **.1470** | **3.89** | **0.000** |
| 2018 | **1.5563** | **.1677** | **4.10** | **0.000** |
| 2019 | **1.7605** | **.1902** | **5.23** | **0.000** |
|  |  |  |  |  |
| Constant | **3.69414** | **.2472** | **19.52** | **0.000** |

**Wald chi2(31) = 1500.03 (p<0.01); N = 37,238**

**
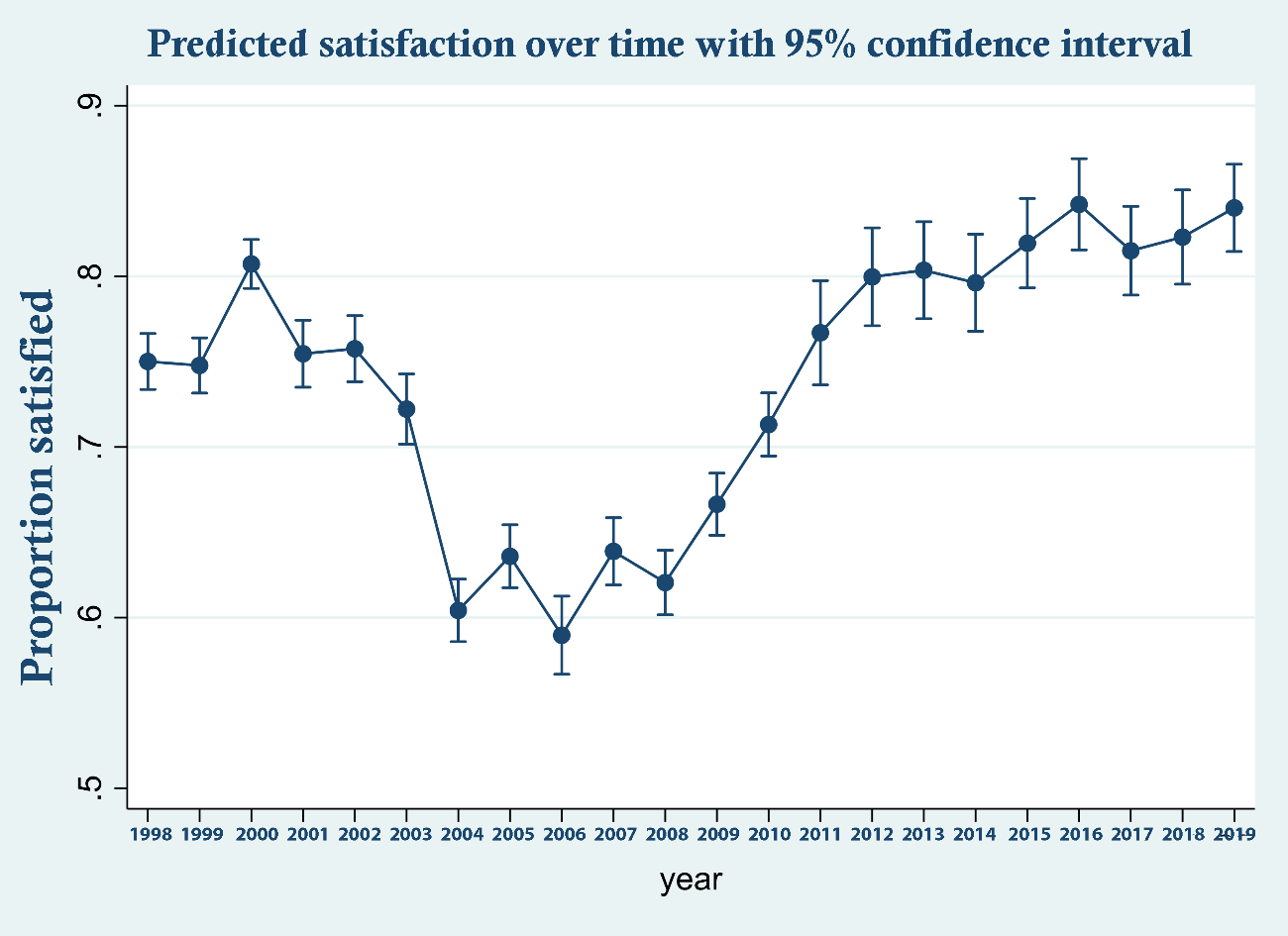
**

**Figure F1 : Satisfaction with publicly funded dental services over time. ( unweighted )**

**Table F2 Predicted satisfaction with publicly funded dental services of who are over 65 versus who are 65 and under. ( unweighted )**

| Independent variable | Odds Ratio | Std. Err. | z | P>\|z\| | |  |
| --- | --- | --- | --- | --- | --- | --- |
|  |  |  |  |  | |  |
| Over 65 | **1.6280** | **.20210** | **3.93** | **0.000** | |  |
|  |  |  |  |  | |  |
| Had a degree | **.7628** | **.0241** | **-8.54** | **0.000** | |  |
|  |  |  |  |  | |  |
| Had Dependent Child in Household | **1.1390** | **.0309** | **4.79** | **0.000** | |  |
|  |  |  |  |  | |  |
| Married | **.9070** | **.0245** | **-3.60** | **0.000** | |  |
|  |  |  |  |  | |  |
| Income Quartile (relative to 1) |  |  |  |  | |  |
| 2 | **.9073** | **.0315** | **-2.79** | **0.005** | |  |
| 3 | **.8668** | **.0323** | **-3.83** | **0.000** | |  |
| 4 | **.7958** | **.0318** | **-5.70** | **0.000** | |  |
|  |  |  |  |  | |  |
| Resides in Scotland | **1.4697** | **.0639** | **8.85** | **0.000** | |  |
|  |  |  |  |  | |  |
| White | **.9024** | **.0412** | **-2.24** | **0.025** | |  |
|  |  |  |  |  | |  |
| Male | **.9609** | **.0231** | **-1.65** | **0.099** | |  |
|  |  |  |  |  | |  |
| Year and Resident in England/Wales (relative to 1998 and living in Scotland) | | | | | |  |
| 1999 | **1.0220** | **.0710** | **0.31** | **0.754** | |  |
| 2000 | **1.4521** | **.1037** | **5.22** | **0.000** | |  |
| 2001 | **1.0320** | **.0799** | **0.41** | **0.684** | |  |
| 2002 | **1.0496** | **.0808** | **0.63** | **0.529** | |  |
| 2003 | **.9471** | **.0725** | **-0.71** | **0.478** | |  |
| 2004 | **.5264** | **.0348** | **-9.70** | **0.000** | |  |
| 2005 | **.5934** | **.0398** | **-7.78** | **0.000** | |  |
| 2006 | **.4804** | **.0353** | **-9.97** | **0.000** | |  |
| 2007 | **.6115** | **.0425** | **-7.07** | **0.000** | |  |
| 2008 | **.5636** | **.0381** | **-8.47** | **0.000** | |  |
| 2009 | **.6886** | **.0467** | **-5.50** | **0.000** | |  |
| 2010 | **.8518** | **.0609** | **-2.24** | **0.025** | |  |
| 2011 | **1.0656** | **.1137** | **0.60** | **0.551** | |  |
| 2012 | **1.4103** | **.1622** | **2.99** | **0.003** | |  |
| 2013 | **1.4011** | **.1596** | **2.96** | **0.003** | |  |
| 2014 | **1.3719** | **.1554** | **2.79** | **0.005** | |  |
| 2015 | **1.5722** | **.1789** | **3.98** | **0.000** | |  |
| 2016 | **1.8499** | **.2335** | **4.87** | **0.000** | |  |
| 2017 | **1.5749** | **.1761** | **4.06** | **0.000** | |  |
| 2018 | **1.5075** | **.1815** | **3.41** | **0.001** | |  |
| 2019 | **1.8044** | **.2161** | **4.93** | **0.000** | |  |
| Year and Resident in Scotland (relative to 1998 and living in England/Wales) | | | | | |  |
| 1999 | **.8333** | **.1277** | **-1.19** | **0.234** | |  |
| 2000 | **1.1659** | **.1923** | **0.93** | **0.352** | |  |
| 2001 | **.9993** | **.1809** | **-0.00** | **0.997** | |  |
| 2002 | **1.0223** | **.1881** | **0.12** | **0.905** | |  |
| 2003 | **.5574** | **.0931** | **-3.50** | **0.000** | |  |
| 2004 | **.4078** | **.0595** | **-6.14** | **0.000** | |  |
| 2005 | **.5047** | **.0759** | **-4.55** | **0.000** | |  |
| 2006 | **.4373** | **.0703** | **-5.14** | **0.000** | |  |
| 2007 | **.4717** | **.0724** | **-4.89** | **0.000** | |  |
| 2008 | **.4403** | **.0657** | **-5.49** | **0.000** | |  |
| 2009 | **.5439** | **.0838** | **-3.95** | **0.000** | |  |
| 2010 | **.7110** | **.1135** | **-2.14** | **0.033** | |  |
| 2011 | **1.4038** | **.4005** | **1.19** | **0.234** | |  |
| 2012 | **1.0381** | **.2390** | **0.16** | **0.871** | |  |
| 2013 | **1.2078** | **.2981** | **0.77** | **0.444** | |  |
| 2014 | **1.0448** | **.2363** | **0.19** | **0.846** | |  |
| 2015 | **1.2824** | **.2943** | **1.08** | **0.278** | |  |
| 2016 | **1.5154** | **.3866** | **1.63** | **0.103** | |  |
| 2017 | **1.1045** | **.2449** | **0.45** | | **0.654** | |
| 2018 | **1.6712** | **.4140** | **2.07** | | **0.038** | |
| 2019 | **1.5498** | **.3900** | **1.74** | | **0.082** | |
|  |  |  |  | |  | |
| Constant | **3.5739** | **.2491** | **18.27** | | **0.000** | |

**Wald chi2(52) = 1506.36 (p<0.01); N = 37,238**

**
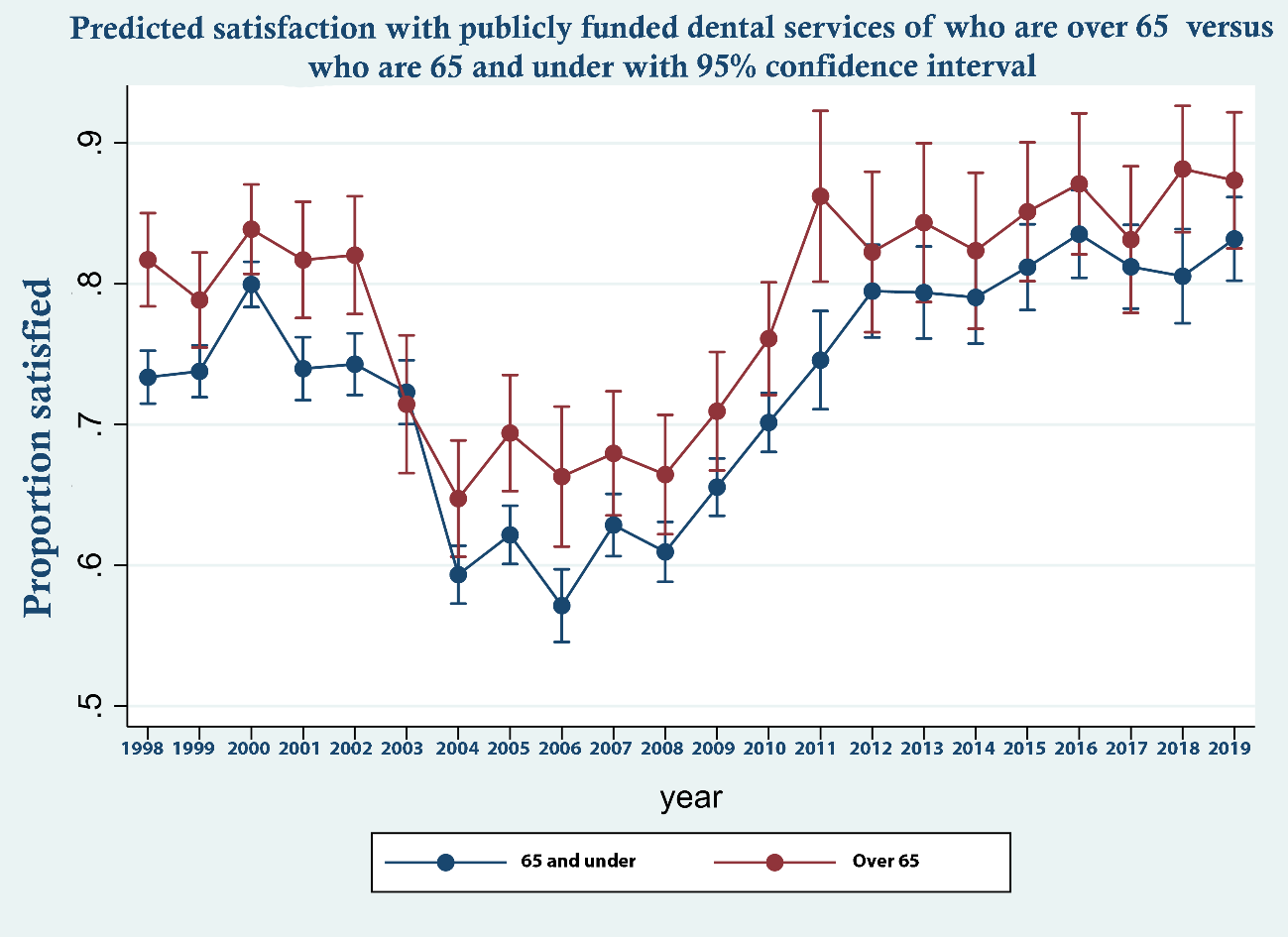
**

**Figure F2: Predicted satisfaction with publicly funded dental services of who are over 65 versus who are 65 and under. ( unweighted )**

**Table F3 Predicted satisfaction with publicly funded dental services of income quartile. ( unweighted )**

| Independent variable | Odds Ratio | Std. Err. | | z | P>\|z |  |
| --- | --- | --- | --- | --- | --- | --- |
|  |  |  | |  |  |  |
| Over 65 | **1.3291** | **.04561** | | **8.29** | **0.000** |  |
|  |  |  | |  |  |  |
| Had a degree | **.7579** | **.0241** | | **-8.71** | **0.000** |  |
|  |  |  | |  |  |  |
| Had Dependent Child in Household | **1.1348** | **.0308** | | **4.65** | **0.000** |  |
|  |  |  | |  |  |  |
| Married | **.9094** | **.0246** | | **-3.50** | **0.000** |  |
|  |  |  | |  |  |  |
| Income Quartile (relative to 1) |  |  | |  |  |  |
| 2 | **.8216** | **.1064** | | **-1.52** | **0.129** |  |
| 3 | **.6096** | **.0783** | | **-3.85** | **0.000** |  |
| 4 | **.7002** | **.0941** | | **-2.65** | **0.008** |  |
|  |  |  | |  |  |  |
| Resides in Scotland | **1.4718** | **.0640** | | **8.88** | **0.000** |  |
|  |  |  | |  |  |  |
| White | **.9042** | **.0413** | | **-2.20** | **0.028** |  |
|  |  |  | |  |  |  |
| Male | **.9619** | **.0232** | | **-1.61** | **0.108** |  |
|  |  |  | |  |  |  |
| Year Relative to Income Quartile |  |  | |  |  |  |
| 1 1999 | **.9592** | **.1273** | | **-0.31** | **0.754** |  |
| 1 2000 | **1.3116** | **.1811** | | **1.96** | **0.050** |  |
| 1 2001 | **.9617** | **.1401** | | **-0.27** | **0.789** |  |
| 1 2002 | **1.0694** | **.1621** | | **0.44** | **0.658** |  |
| 1 2003 | **.7286** | **.1048** | | **-2.20** | **0.028** |  |
| 1 2004 | **.4718** | **.0566** | | **-6.25** | **0.000** |  |
| 1 2005 | **.5758** | **.0704** | | **-4.51** | **0.000** |  |
| 1 2006 | **.4916** | **.0676** | | **-5.16** | **0.000** |  |
| 1 2007 | **.4687** | **.0586** | | **-6.05** | **0.000** |  |
| 1 2008 | **.4791** | **.0598** | | **-5.89** | **0.000** |  |
| 1 2009 | **.4637** | **.0588** | | **-6.05** | **0.000** |  |
| 1 2010 | **.6362** | **.0810** | | **-3.55** | **0.000** |  |
| 1 2011 | **.6889** | **.1274** | | **-2.01** | **0.044** |  |
| 1 2012 | **.9911** | **.1944** | | **-0.05** | **0.964** |  |
| 1 2013 | **.7983** | **.1488** | | **-1.21** | **0.227** |  |
| 1 2014 | **1.0723** | **.2124** | | **0.35** | **0.724** |  |
| 1 2015 | **1.0981** | **.2139** | | **0.48** | **0.631** |  |
| 1 2016 | **1.0617** | **.2505** | | **0.25** | **0.800** |  |
| 1 2017 | **1.1721** | **.2529** | | **0.74** | **0.462** |  |
| 1 2018 | **1.0197** | **.2048** | | **0.10** | **0.922** |  |
| 1 2019 | **1.2344** | **.2619** | | **0.99** | **0.321** |  |
|  |  |  | |  |  |  |
| 2 1999 | **.9127** | **.1120** | | **-0.74** | **0.457** |  |
| 2 2000 | **1.4366** | **.1858** | | **2.80** | **0.005** |  |
| 2 2001 | **.9986** | **.1455** | | **-0.01** | **0.993** |  |
| 2 2002 | **.9883** | **.1417** | | **-0.08** | **0.935** |  |
| 2 2003 | **.8225** | **.1148** | | **-1.40** | **0.162** |  |
| 2 2004 | **.4825** | **.0580** | | **-6.06** | **0.000** |  |
| 2 2005 | **.4717** | **.0577** | | **-6.14** | **0.000** |  |
| 2 2006 | **.3937** | **.0504** | | **-7.27** | **0.000** |  |
| 2 2007 | **.6226** | **.0828** | | **-3.56** | **0.000** |  |
| 2 2008 | **.4888** | **.0590** | | **-5.93** | **0.000** |  |
| 2 2009 | **.6856** | **.0826** | | **-3.13** | **0.002** |  |
| 2 2010 | **.8066** | **.1080** | | **-1.60** | **0.109** |  |
| 2 2011 | **1.1459** | **.2369** | | **0.66** | **0.510** |  |
| 2 2012 | **1.0318** | **.2114** | | **0.15** | **0.878** |  |
| 2 2013 | **1.7161** | **.4049** | | **2.29** | **0.022** |  |
| 2 2014 | **1.3960** | **.2980** | | **1.56** | **0.118** |  |
| 2 2015 | **1.5819** | **.3536** | | **2.05** | **0.040** |  |
| 2 2016 | **1.4645** | **.3099** | | **1.80** | **0.071** |  |
| 2 2017 | **1.6843** | **.3591** | | **2.44** | **0.014** |  |
| 2 2018 | **1.3906** | **.2845** | **1.61** | | **0.107** | |
| 2 2019 | **1.4548** | **.3095** | **1.76** | | **0.078** | |
|  |  |  |  | |  | |
| 3 1999 | **1.0980** | **.1348** | **0.76** | | **0.446** | |
| 3 2000 | **1.6537** | **.2106** | **3.95** | | **0.000** | |
| 3 2001 | **1.2415** | **.1696** | **1.58** | | **0.113** | |
| 3 2002 | **1.2314** | **.1656** | **1.55** | | **0.122** | |
| 3 2003 | **1.0358** | **.1347** | **0.27** | | **0.787** | |
| 3 2004 | **.5327** | **.0634** | **-5.29** | | **0.000** | |
| 3 2005 | **.6751** | **.0828** | **-3.20** | | **0.001** | |
| 3 2006 | **.6160** | **.0794** | **-3.76** | | **0.000** | |
| 3 2007 | **.7129** | **.0856** | **-2.82** | | **0.005** | |
| 3 2008 | **.7507** | **.0916** | **-2.35** | | **0.019** | |
| 3 2009 | **.9668** | **.1206** | **-0.27** | | **0.787** | |
| 3 2010 | **1.0755** | **.1395** | **0.56** | | **0.575** | |
| 3 2011 | **1.6148** | **.3322** | **2.33** | | **0.020** | |
| 3 2012 | **1.8870** | **.3889** | **3.08** | | **0.002** | |
| 3 2013 | **1.7295** | **.3626** | **2.61** | | **0.009** | |
| 3 2014 | **1.6892** | **.3490** | **2.54** | | **0.011** | |
| 3 2015 | **2.0566** | **.4405** | **3.37** | | **0.001** | |
| 3 2016 | **1.9446** | **.4227** | **3.06** | | **0.002** | |
| 3 2017 | **1.8557** | **.3782** | **3.03** | | **0.002** | |
| 3 2018 | **2.0943** | **.4666** | **3.32** | | **0.001** | |
| 3 2019 | **3.5336** | **.8545** | **5.22** | | **0.000** | |
|  |  |  |  | |  | |
| 4 1999 | **.9921** | **.1289** | **-0.06** | | **0.952** | |
| 4 2000 | **1.2315** | **.1631** | **1.57** | | **0.116** | |
| 4 2001 | **.9222** | **.1323** | **-0.56** | | **0.573** | |
| 4 2002 | **.9221** | **.1310** | **-0.57** | | **0.569** | |
| 4 2003 | **.8982** | **.1308** | **-0.74** | | **0.461** | |
| 4 2004 | **.5172** | **.0644** | **-5.29** | | **0.000** | |
| 4 2005 | **.5822** | **.0728** | **-4.33** | | **0.000** | |
| 4 2006 | **.4187** | **.0602** | **-6.05** | | **0.000** | |
| 4 2007 | **.5758** | **.0752** | **-4.22** | | **0.000** | |
| 4 2008 | **.4841** | **.0611** | **-5.74** | | **0.000** | |
| 4 2009 | **.6181** | **.0776** | **-3.83** | | **0.000** | |
| 4 2010 | **.8540** | **.1121** | **-1.20** | | **0.230** | |
| 4 2011 | **1.1925** | **.2354** | **0.89** | | **0.373** | |
| 4 2012 | **1.6366** | **.3497** | **2.31** | | **0.021** | |
| 4 2013 | **1.6252** | **.3315** | **2.38** | | **0.017** | |
| 4 2014 | **1.1751** | **.2297** | **0.83** | | **0.409** | |
| 4 2015 | **1.5379** | **.2920** | **2.27** | | **0.023** | |
| 4 2016 | **3.0295** | **.7190** | **4.67** | | **0.000** | |
| 4 2017 | **1.3299** | **.2378** | **1.59** | | **0.111** | |
| 4 2018 | **2.1139** | **.4958** | **3.19** | | **0.001** | |
| 4 2019 | **1.5926** | **.3282** | **2.26** | | **0.024** | |
|  |  |  |  | |  | |
| Constant | **4.2771** | **.4443** | **13.99** | | **0.000** | |

**Wald chi2(94) = 1602.94 (p<0.01); N = 37,238**

**
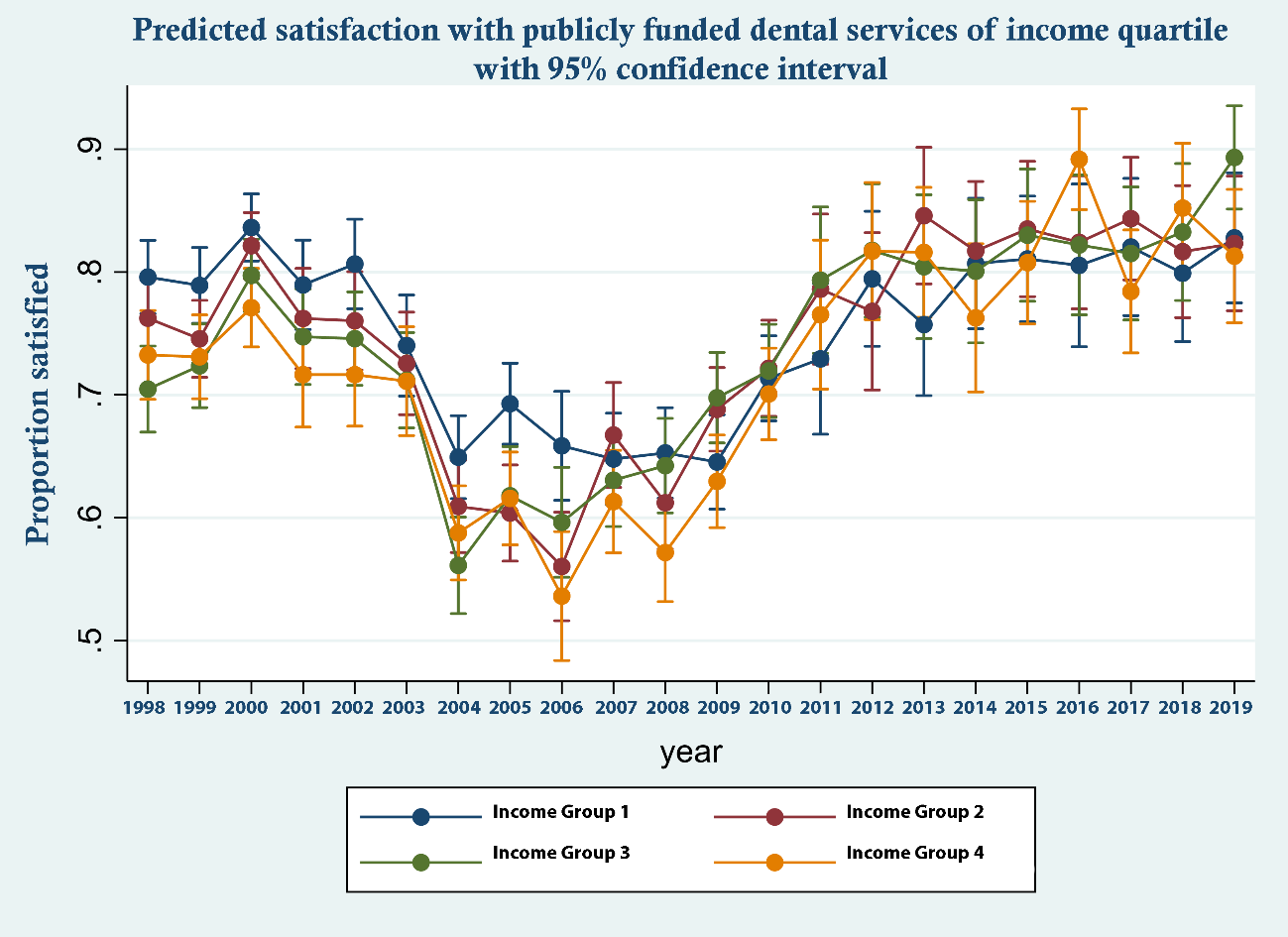
**

**Figure F3: Predicted satisfaction with publicly funded dental services of income quartile. ( unweighted )**

**Table F4 Predicted satisfaction with publicly funded dental services of household who have dependent child versus who don’t . ( unweighted )**

| Independent variable | Odds Ratio | Std. Err. | z | | P>\|z |
| --- | --- | --- | --- | --- | --- |
|  |  |  |  |  | |
| Over 65 | **1.3485** | **.04614** | **8.74** | **0.000** | |
|  |  |  |  |  | |
| Had a degree | **.7629** | **.02419** | **-8.53** | **0.000** | |
|  |  |  |  |  | |
| Had Dependent Child in Household | **1.0722** | **.10608** | **0.71** | **0.481** | |
|  |  |  |  |  | |
| Married | **.9067** | **.02455** | **-3.62** | **0.000** | |
|  |  |  |  |  | |
| Income Quartile (relative to 1) |  |  |  |  | |
| 2 | **.9066** | **.03148** | **-2.82** | **0.005** | |
| 3 | **.8666** | **.03231** | **-3.84** | **0.000** | |
| 4 | **.7968** | **.03187** | **-5.68** | **0.000** | |
|  |  |  |  |  | |
| Resides in Scotland | **1.4677** | **.06391** | **8.81** | **0.000** | |
|  |  |  |  |  | |
| White | **.9027** | **.04127** | **-2.24** | **0.025** | |
|  |  |  |  |  | |
| Male | **.9611** | **.02318** | **-1.64** | **0.100** | |
|  |  |  |  |  | |
| Year and Resident in England/Wales (relative to 1998 and living in Scotland) | | | | | |
| 1999 | **.9769** | **.0733** | **-0.31** | **0.756** | |
| 2000 | **1.3800** | **.1074** | **4.14** | **0.000** | |
| 2001 | **1.0018** | **.0854** | **0.02** | **0.983** | |
| 2002 | **1.0835** | **.0942** | **0.92** | **0.356** | |
| 2003 | **.7908** | **.0661** | **-2.80** | **0.005** | |
| 2004 | **.4917** | **.0353** | **-9.88** | **0.000** | |
| 2005 | **.5720** | **.0417** | **-7.66** | **0.000** | |
| 2006 | **.4688** | **.0372** | **-9.54** | **0.000** | |
| 2007 | **.5690** | **.0435** | **-7.37** | **0.000** | |
| 2008 | **.5318** | **.0402** | **-8.34** | **0.000** | |
| 2009 | **.6159** | **.0461** | **-6.46** | **0.000** | |
| 2010 | **.8052** | **.0649** | **-2.69** | **0.007** | |
| 2011 | **1.0635** | **.1335** | **0.49** | **0.624** | |
| 2012 | **1.5024** | **.2023** | **3.02** | **0.003** | |
| 2013 | **1.3305** | **.1689** | **2.25** | **0.024** | |
| 2014 | **1.2810** | **.1615** | **1.96** | **0.050** | |
| 2015 | **1.5829** | **.1998** | **3.64** | **0.000** | |
| 2016 | **1.9369** | **.2787** | **4.59** | **0.000** | |
| 2017 | **1.3973** | **.1703** | **2.74** | **0.006** | |
| 2018 | **1.5667** | **.2111** | **3.33** | **0.001** | |
| 2019 | **1.7613** | **.2379** | **4.19** | **0.000** | |
| Year and Resident in Scotland (relative to 1998 and living in England/Wales) | | | | | |
| 1999 | **1.0120** | **.1179** | **0.10** | **0.918** | |
| 2000 | **1.4489** | **.1750** | **3.07** | **0.002** | |
| 2001 | **1.0812** | **.1383** | **0.61** | **0.542** | |
| 2002 | **.9773** | **.1196** | **-0.19** | **0.852** | |
| 2003 | **1.0505** | **.1323** | **0.39** | **0.696** | |
| 2004 | **.5337** | **.0585** | **-5.73** | **0.000** | |
| 2005 | **.5912** | **.0661** | **-4.70** | **0.000** | |
| 2006 | **.4860** | **.0595** | **-5.89** | **0.000** | |
| 2007 | **.6229** | **.0700** | **-4.21** | **0.000** | |
| 2008 | **.5620** | **.0597** | **-5.42** | **0.000** | |
| 2009 | **.7673** | **.0847** | **-2.40** | **0.016** | |
| 2010 | **.8730** | **.0972** | **-1.22** | **0.223** | |
| 2011 | **1.1690** | **.1903** | **0.96** | **0.337** | |
| 2012 | **1.1474** | **.1862** | **0.85** | **0.397** | |
| 2013 | **1.4530** | **.2584** | **2.10** | **0.036** | |
| 2014 | **1.3656** | **.2319** | **1.83** | **0.067** | |
| 2015 | **1.4233** | **.2458** | **2.04** | **0.041** | |
| 2016 | **1.5926** | **.29330** | **2.53** | **0.011** | |
| 2017 | **1.6446** | **.28540** | **2.87** | **0.004** | |
| 2018 | **1.5573** | **.27995** | **2.46** | **0.014** | |
| 2019 | **1.7796** | **.32118** | **3.19** | **0.001** | |
|  |  |  |  |  | |
| Constant | **3.754775** | **.2725** | **18.23** | **0.000** | |

**Wald chi2(52) = 1513.84 (p<0.01); N = 37,238**

**
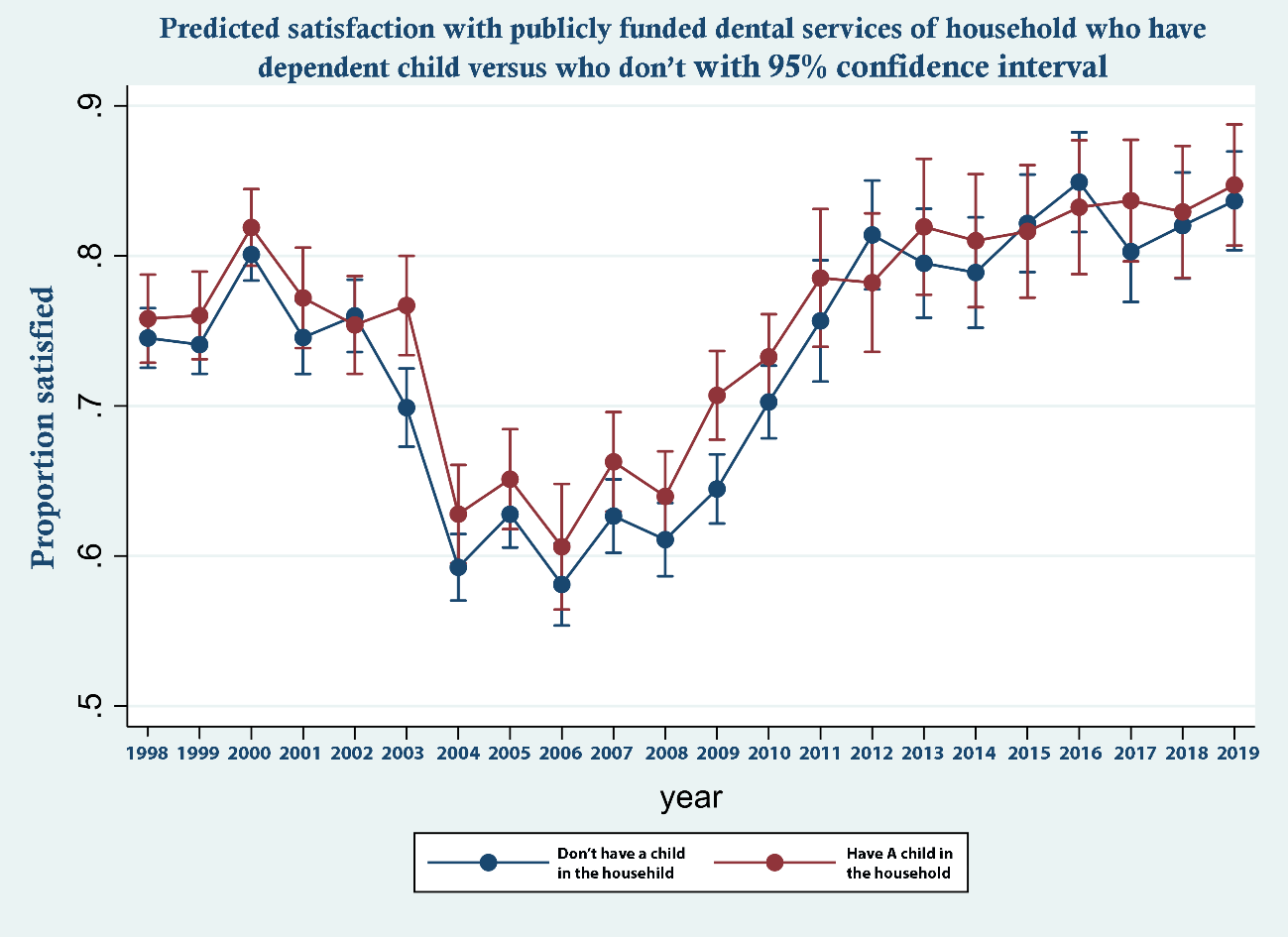
**

**Figure F4 : Predicted satisfaction with publicly funded dental services of household who have dependent child versus who don’t . ( unweighted )**

**Table F5 Predicted satisfaction with publicly funded dental services of those who are white versus who are not over time. ( unweighted )**

| Independent variable | Odds Ratio | Std. Err. | z | P>\|z\| | |
| --- | --- | --- | --- | --- | --- |
| Over 65 | **1.3482** | **.0461** | **8.73** | **0.000** | |
|  |  |  |  |  | |
| Had a Degree | **.7633** | **.0242** | **-8.51** | **0.000** | |
|  |  |  |  |  | |
| Had Dependent Child in Household | **1.1411** | **.0310** | **4.86** | **0.000** | |
|  |  |  |  |  | |
| Married | **.9066** | **.0245** | **-3.62** | **0.000** | |
|  |  |  |  |  | |
| Income Quartile (relative to 1) |  |  |  |  | |
| 2 | **.9058** | **.03145** | **-2.85** | **0.004** | |
| 3 | **.8660** | **.03225** | **-3.86** | **0.000** | |
| 4 | **.7935** | **.03173** | **-5.78** | **0.000** | |
|  |  |  |  |  | |
| Resides in Scotland | **1.4704** | **.0640** | **8.85** | **0.000** | |
|  |  |  |  |  | |
| White | **.8172** | **.1580** | **-1.04** | **0.297** | |
|  |  |  |  |  | |
| Male | **.9616** | **.0231** | **-1.62** | **0.105** | |
|  |  |  |  |  | |
| Year and Resident in England/Wales (relative to 1998 and living in Scotland) | | | | | |
| 1999 | **1.1961** | **.3416** | **0.63** | **0.531** | |
| 2000 | **1.3496** | **.3545** | **1.14** | **0.254** | |
| 2001 | **.7565** | **.2128** | **-0.99** | **0.321** | |
| 2002 | **.6153** | **.1593** | **-1.88** | **0.061** | |
| 2003 | **.6504** | **.1668** | **-1.68** | **0.094** | |
| 2004 | **.5933** | **.1527** | **-2.03** | **0.043** | |
| 2005 | **.7232** | **.1785** | **-1.31** | **0.189** | |
| 2006 | **.4421** | **.1130** | **-3.19** | **0.001** | |
| 2007 | **.6717** | **.1609** | **-1.66** | **0.097** | |
| 2008 | **.5024** | **.1182** | **-2.92** | **0.003** | |
| 2009 | **.6951** | **.1678** | **-1.51** | **0.132** | |
| 2010 | **.6153** | **.1531** | **-1.95** | **0.051** | |
| 2011 | **.9879** | **.3676** | **-0.03** | **0.974** | |
| 2012 | **.9699** | **.3491** | **-0.08** | **0.932** | |
| 2013 | **1.0412** | **.3389** | **0.12** | **0.901** | |
| 2014 | **1.1518** | **.3987** | **0.41** | **0.683** | |
| 2015 | **.9575** | **.3171** | **-0.13** | **0.896** | |
| 2016 | **.9463** | **.3569** | **-0.15** | **0.884** | |
| 2017 | **1.2250** | **.4148** | **0.60** | **0.549** | |
| 2018 | **1.3708** | **.4875** | **0.89** | **0.375** | |
| 2019 | **1.0298** | **.3610** | **0.08** | **0.933** | |
| Year and Resident in Scotland (relative to 1998 and living in England/Wales) | | | | | |
| 1999 | **.9800** | **.0635** | **-0.31** | **0.756** | |
| 2000 | **1.4032** | **.0948** | **5.01** | **0.000** | |
| 2001 | **1.0465** | **.0768** | **0.62** | **0.535** | |
| 2002 | **1.0914** | **.0805** | **1.19** | **0.236** | |
| 2003 | **.8859** | **.0642** | **-1.67** | **0.095** | |
| 2004 | **.4996** | **.0309** | **-11.21** | **0.000** | |
| 2005 | **.5679** | **.0358** | **-8.96** | **0.000** | |
| 2006 | **.4757** | **.0328** | **-10.75** | **0.000** | |
| 2007 | **.5751** | **.0377** | **-8.42** | **0.000** | |
| 2008 | **.5424** | **.0346** | **-9.59** | **0.000** | |
| 2009 | **.6579** | **.0422** | **-6.52** | **0.000** | |
| 2010 | **.8457** | **.0571** | **-2.48** | **0.013** | |
| 2011 | **1.1050** | **.1136** | **0.97** | **0.332** | |
| 2012 | **1.3699** | **.1473** | **2.93** | **0.003** | |
| 2013 | **1.4092** | **.1544** | **3.13** | **0.002** | |
| 2014 | **1.3180** | **.1396** | **2.61** | **0.009** | |
| 2015 | **1.5924** | **.1711** | **4.33** | **0.000** | |
| 2016 | **1.8970** | **.2256** | **5.38** | **0.000** | |
| 2017 | **1.4976** | **.1567** | **3.86** | **0.000** | |
| 2018 | **1.5717** | **.1780** | **3.99** | **0.000** | |
| 2019 | **1.8578** | **.2118** | **5.43** | | **0.000** |
|  |  |  |  | |  |
| Constant | **4.0516** | **.7663** | **7.40** | | **0.000** |

**Wald chi2(52) = 1528.04 (p<0.01); N = 37,238**


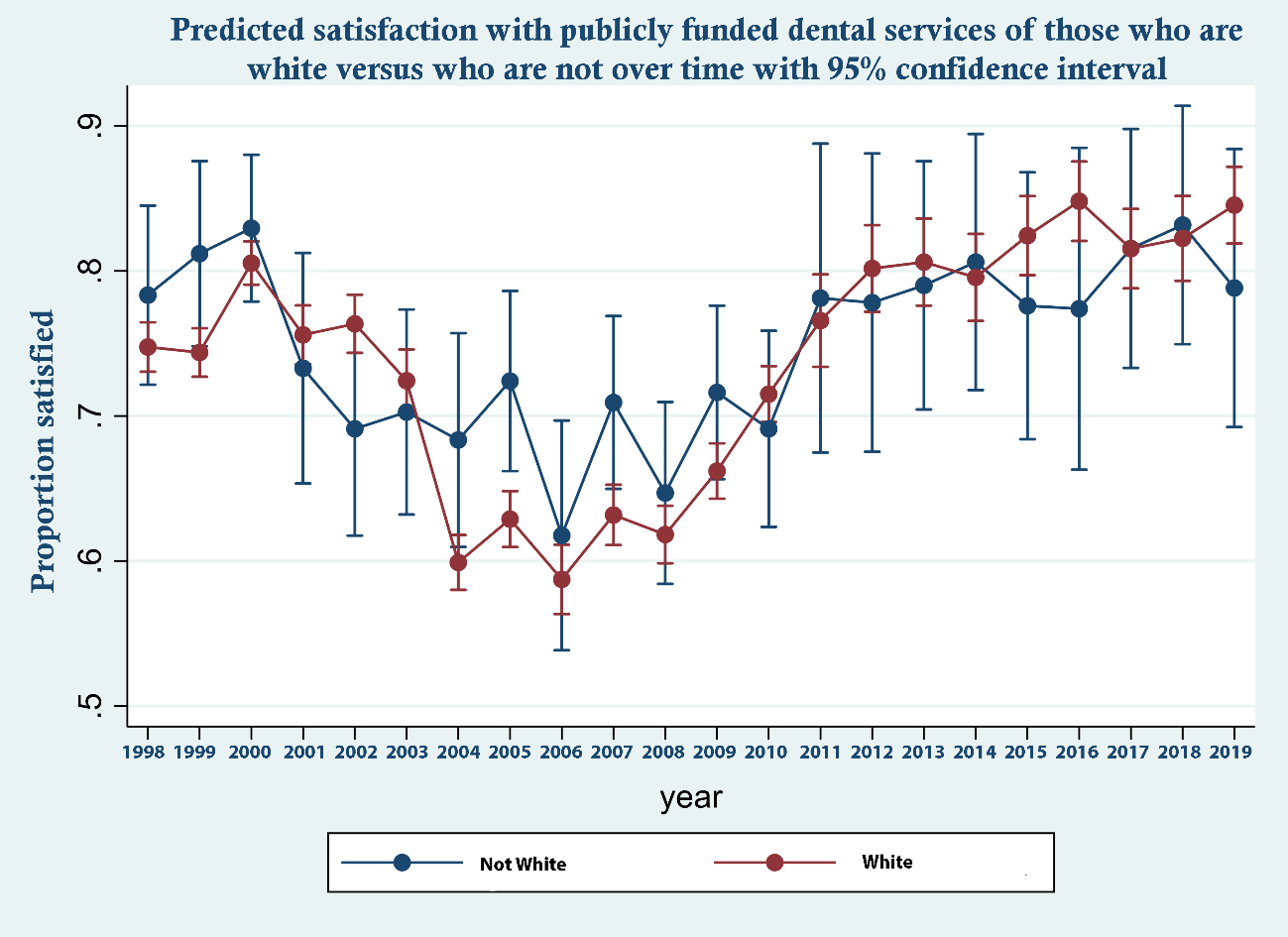


**Figure F5 : Predicted satisfaction with publicly funded dental services of those who are white versus who are not over time. ( unweighted )**

**Table F6 Predicted satisfaction with publicly funded dental services of those who have a degree versus who don’t. ( unweighted )**

| Independent variable | Odds Ratio | Std. Err. | z | P>\|z\| |
| --- | --- | --- | --- | --- |
| Over 65 | **1.3483** | **.0460** | **8.75** | **0.000** |
|  |  |  |  |  |
| Had a degree | **.6618** | **.0950** | **-2.87** | **0.004** |
|  |  |  |  |  |
| Had Dependent Child in Household | **1.1392** | **.0309** | **4.80** | **0.000** |
|  |  |  |  |  |
| Married | **.9063** | **.0245** | **-3.63** | **0.000** |
|  |  |  |  |  |
|  |  |  |  |  |
| Income Quartile (relative to 1) |  |  |  |  |
| 2 | **.9051** | **.0314** | **-2.87** | **0.004** |
| 3 | **.8665** | **.0322** | **-3.84** | **0.000** |
| 4 | **.7973** | **.0319** | **-5.66** | **0.000** |
|  |  |  |  |  |
| Resides in Scotland | **1.4706** | **.0639** | **8.87** | **0.000** |
|  |  |  |  |  |
| White | **.9012** | **.0412** | **-2.27** | **0.023** |
|  |  |  |  |  |
| Male | **.9616** | **.0232** | **-1.62** | **0.105** |
|  |  |  |  |  |
| Year and Resident in England/Wales (relative to 1998 and living in Scotland) | | | | |
| 1999 | **1.0046** | **.0681** | **0.07** | **0.946** |
| 2000 | **1.4079** | **.0999** | **4.82** | **0.000** |
| 2001 | **1.0023** | **.0776** | **0.03** | **0.976** |
| 2002 | **1.0138** | **.0779** | **0.18** | **0.858** |
| 2003 | **.8160** | **.0613** | **-2.70** | **0.007** |
| 2004 | **.4898** | **.0317** | **-11.00** | **0.000** |
| 2005 | **.5853** | **.0387** | **-8.09** | **0.000** |
| 2006 | **.4556** | **.0330** | **-10.85** | **0.000** |
| 2007 | **.5747** | **.0399** | **-7.97** | **0.000** |
| 2008 | **.5488** | **.0369** | **-8.91** | **0.000** |
| 2009 | **.6474** | **.0441** | **-6.38** | **0.000** |
| 2010 | **.7850** | **.0566** | **-3.35** | **0.001** |
| 2011 | **1.0627** | **.1175** | **0.55** | **0.582** |
| 2012 | **1.2527** | **.1441** | **1.96** | **0.050** |
| 2013 | **1.3380** | **.1601** | **2.43** | **0.015** |
| 2014 | **1.3001** | **.1519** | **2.25** | **0.025** |
| 2015 | **1.5975** | **.1920** | **3.90** | **0.000** |
| 2016 | **1.6304** | **.2138** | **3.73** | **0.000** |
| 2017 | **1.5422** | **.1842** | **3.63** | **0.000** |
| 2018 | **1.3640** | **.1671** | **2.53** | **0.011** |
| 2019 | **1.7326** | **.2212** | **4.30** | **0.000** |
| Year and Resident in Scotland (relative to 1998 and living in England/Wales) | | | | |
| 1999 | **.9189** | **.1641** | **-0.47** | **0.636** |
| 2000 | **1.4349** | **.2531** | **2.05** | **0.041** |
| 2001 | **1.1969** | **.2209** | **0.97** | **0.330** |
| 2002 | **1.2431** | **.2313** | **1.17** | **0.242** |
| 2003 | **1.1993** | **.2233** | **0.98** | **0.329** |
| 2004 | **.6058** | **.0996** | **-3.05** | **0.002** |
| 2005 | **.5719** | **.0956** | **-3.34** | **0.001** |
| 2006 | **.5941** | **.1039** | **-2.98** | **0.003** |
| 2007 | **.6688** | **.1093** | **-2.46** | **0.014** |
| 2008 | **.5463** | **.0888** | **-3.72** | **0.000** |
| 2009 | **.7677** | **.1234** | **-1.64** | **0.100** |
| 2010 | **1.0381** | **.1696** | **0.23** | **0.819** |
| 2011 | **1.3181** | **.3046** | **1.20** | **0.232** |
| 2012 | **1.7639** | **.4158** | **2.41** | **0.016** |
| 2013 | **1.5748** | **.3464** | **2.06** | **0.039** |
| 2014 | **1.4383** | **.3134** | **1.67** | **0.095** |
| 2015 | **1.4685** | **.3134** | **1.80** | **0.072** |
| 2016 | **2.4201** | **.5675** | **3.77** | **0.000** |
| 2017 | **1.4678** | **.3015** | **1.87** | **0.062** |
| 2018 | **2.3532** | **.5466** | **3.68** | **0.000** |
| 2019 | **1.9987** | **.4413** | **3.14** | **0.002** |
|  |  |  |  |  |
| Constant | **3.752513** | **.258397** | **19.20** | **0.000** |

**Wald chi2(52) = 1524.42 (p<0.01); N = 37,238**

**
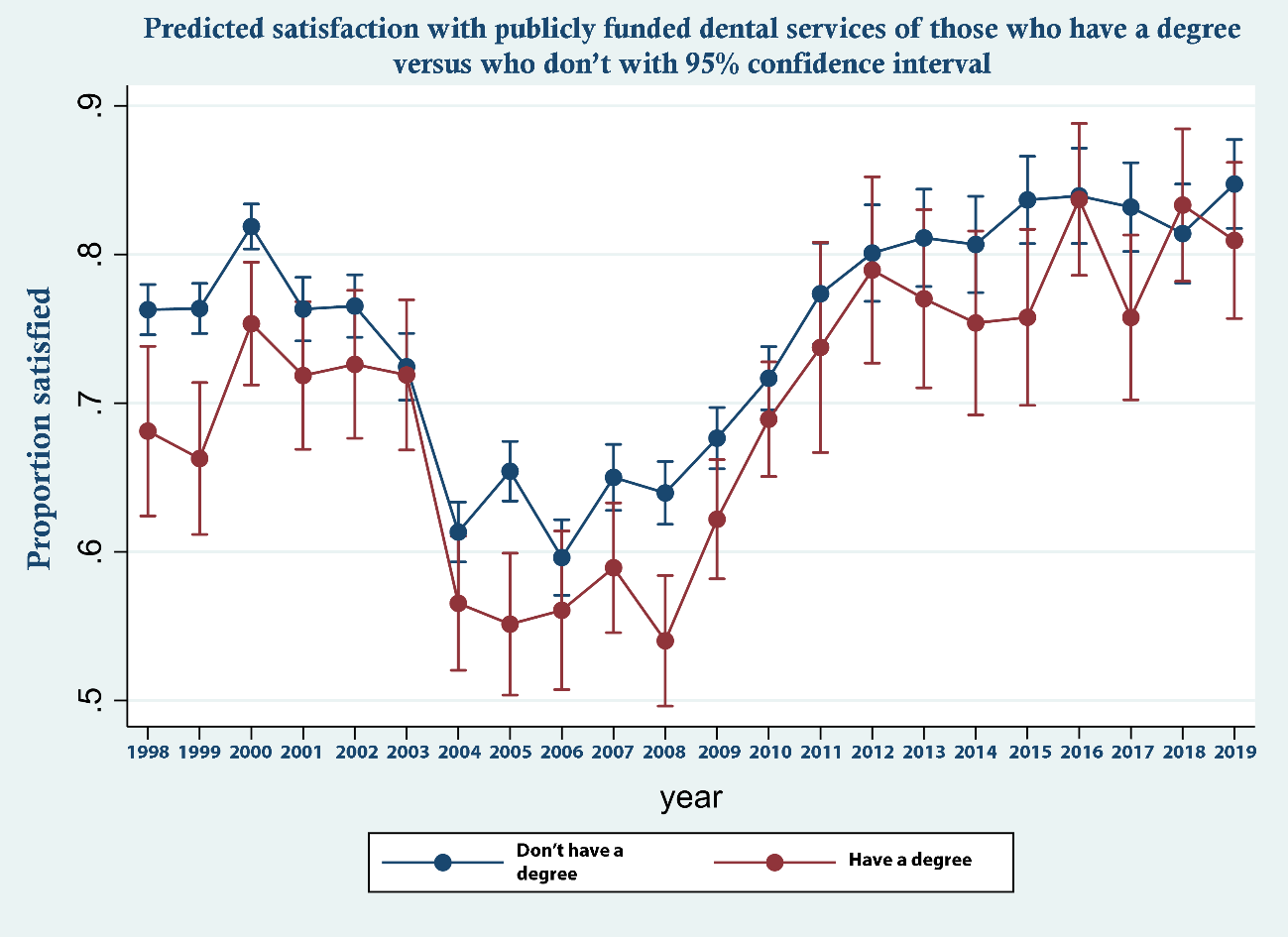
**

**Figure F6: Predicted satisfaction with publicly funded dental services of those who have a degree versus who don’t. ( unweighted )**

**Table F7 Predicted satisfaction with publicly funded dental services of male versus female. ( unweighted )**

|  | | | | |
| --- | --- | --- | --- | --- |
| Independent variable | **Odds Ratio** | **Std. Err.** | **z** | **P>\|z** |
| Over65 | **1.3463** | **.0460** | **8.69** | **0.000** |
|  |  |  |  |  |
| Had a degree | **.7634** | **.0242** | **-8.51** | **0.000** |
|  |  |  |  |  |
| Had Dependent Child in Household | **1.1398** | **.0309** | **4.82** | **0.000** |
| Married | **.9056** | **.0245** | **-3.66** | **0.000** |
|  |  |  |  |  |
| Income Quartile (relative to 1) |  |  |  |  |
| 2 | **.9065** | **.0314** | **-2.83** | **0.005** |
| 3 | **.8674** | **.0323** | **-3.81** | **0.000** |
| 4 | **.7968** | **.0318** | **-5.67** | **0.000** |
|  |  |  |  |  |
| Resides in Scotland | **1.4679** | **.0639** | **8.81** | **0.000** |
|  |  |  |  |  |
| White | **.9032** | **.0413** | **-2.23** | **0.026** |
|  |  |  |  |  |
| Male | **.7959** | **.0722** | **-2.51** | **0.012** |
|  |  |  |  |  |
| Year and Resident in England/Wales (relative to 1998 and living in Scotland) | | | | |
| 1999 | **.9805** | **.0853** | **-0.23** | **0.821** |
| 2000 | **1.3539** | **.1209** | **3.39** | **0.001** |
| 2001 | **.9115** | **.0873** | **-0.97** | **0.334** |
| 2002 | **.9643** | **.0929** | **-0.38** | **0.707** |
| 2003 | **.7944** | **.0747** | **-2.45** | **0.014** |
| 2004 | **.4771** | **.0388** | **-9.09** | **0.000** |
| 2005 | **.5522** | **.0459** | **-7.14** | **0.000** |
| 2006 | **.4233** | **.0383** | **-9.48** | **0.000** |
| 2007 | **.5390** | **.0459** | **-7.24** | **0.000** |
| 2008 | **.4829** | **.0397** | **-8.85** | **0.000** |
| 2009 | **.6141** | **.0515** | **-5.81** | **0.000** |
| 2010 | **.6925** | **.0599** | **-4.24** | **0.000** |
| 2011 | **1.0199** | **.1372** | **0.15** | **0.883** |
| 2012 | **1.1148** | **.1549** | **0.78** | **0.434** |
| 2013 | **1.0978** | **.1478** | **0.69** | **0.488** |
| 2014 | **1.2244** | **.1722** | **1.44** | **0.150** |
| 2015 | **1.1799** | **.1560** | **1.25** | **0.211** |
| 2016 | **1.3551** | **.1940** | **2.12** | **0.034** |
| 2017 | **1.4903** | **.2096** | **2.84** | **0.005** |
| 2018 | **1.4067** | **.2050** | **2.34** | **0.019** |
| 2019 | **1.4231** | **.2017** | **2.49** | **0.013** |
| Year and Resident in Scotland (relative to 1998 and living in England/Wales) | | | | |
| 1999 | **1.0055** | **.0927** | **0.06** | **0.952** |
| 2000 | **1.4612** | **.1409** | **3.93** | **0.000** |
| 2001 | **1.1825** | **.1252** | **1.58** | **0.113** |
| 2002 | **1.1443** | **.1194** | **1.29** | **0.196** |
| 2003 | **.9586** | **.0994** | **-0.41** | **0.684** |
| 2004 | **.5380** | **.0481** | **-6.93** | **0.000** |
| 2005 | **.6112** | **.0552** | **-5.45** | **0.000** |
| 2006 | **.5437** | **.0534** | **-6.20** | **0.000** |
| 2007 | **.6465** | **.0609** | **-4.62** | **0.000** |
| 2008 | **.6224** | **.0577** | **-5.11** | **0.000** |
| 2009 | **.7252** | **.0669** | **-3.48** | **0.000** |
| 2010 | **1.0392** | **.1028** | **0.39** | **0.697** |
| 2011 | **1.2000** | **.1760** | **1.24** | **0.214** |
| 2012 | **1.6495** | **.2518** | **3.28** | **0.001** |
| 2013 | **1.8154** | **.2925** | **3.70** | **0.000** |
| 2014 | **1.4172** | **.2063** | **2.40** | **0.017** |
| 2015 | **2.1089** | **.3373** | **4.66** | **0.000** |
| 2016 | **2.6822** | **.500** | **5.28** | **0.000** |
| 2017 | **1.4748** | **.2095** | **2.74** | **0.006** |
| 2018 | **1.7602** | **.2809** | **3.54** | **0.000** |
| 2019 | **2.3019** | **.3839** | **5.00** | **0.000** |
|  |  |  |  |  |
| Constant | **4.0145** | **.3152** | **17.70** | **0.000** |

**Wald chi2(52) = 1527.26 (p<0.01); N = 37,238**

**
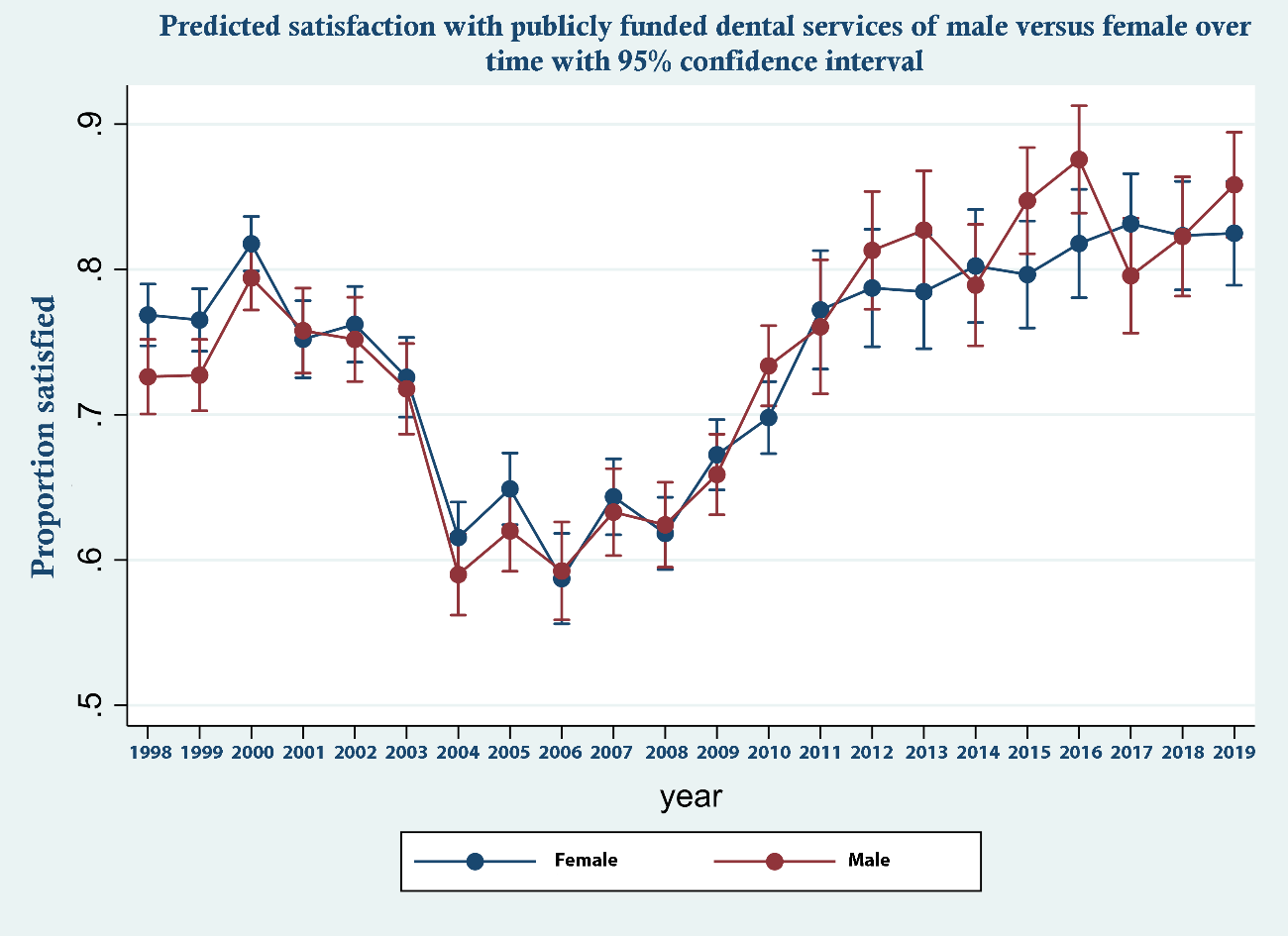
**

**Figure F7: Predicted satisfaction with publicly funded dental services of male versus female.**

**(unweighted)**

**Table F8 Predicted satisfaction with publicly funded dental services of married versus who are not.**

**( unweighted )**

| Independent variable | Odds Ratio | Std. Err. | z | P>\|z\| |
| --- | --- | --- | --- | --- |
| Over 65 | **1.3463** | **.0460** | **8.69** | **0.000** |
|  |  |  |  |  |
| Had a degree | **.7607** | **.0241** | **-8.62** | **0.000** |
|  |  |  |  |  |
| Had Dependent Child in Household | **1.1375** | **.0309** | **4.74** | **0.000** |
|  |  |  |  |  |
| Married | **.8781** | **.0816** | **-1.40** | **0.163** |
|  |  |  |  |  |
| Income Quartile (relative to 1) |  |  |  |  |
| 2 | **.9072** | **.0315** | **-2.80** | **0.005** |
| 3 | **.8676** | **.0323** | **-3.81** | **0.000** |
| 4 | **.7964** | **.0318** | **-5.69** | **0.000** |
|  |  |  |  |  |
| Resides in Scotland | **1.4686** | **.0639** | **8.83** | **0.000** |
|  |  |  |  |  |
| White | **.9018** | **.0412** | **-2.26** | **0.024** |
|  |  |  |  |  |
| Male | **.9618** | **.0232** | **-1.61** | **0.107** |
|  |  |  |  |  |
| Year and Resident in England/Wales (relative to 1998 and living in Scotland) | | | | |
| 1999 | **.9795** | **.0970** | **-0.21** | **0.835** |
| 2000 | **1.2935** | **.1312** | **2.54** | **0.011** |
| 2001 | **1.0869** | **.1201** | **0.75** | **0.451** |
| 2002 | **1.0952** | **.1223** | **0.81** | **0.416** |
| 2003 | **.8709** | **.0938** | **-1.28** | **0.200** |
| 2004 | **.5324** | **.0500** | **-6.70** | **0.000** |
| 2005 | **.5827** | **.0561** | **-5.61** | **0.000** |
| 2006 | **.4664** | **.0490** | **-7.25** | **0.000** |
| 2007 | **.5508** | **.0535** | **-6.14** | **0.000** |
| 2008 | **.5714** | **.0548** | **-5.83** | **0.000** |
| 2009 | **.5714** | **.0548** | **-5.83** | **0.000** |
| 2010 | **.7774** | **.0783** | **-2.50** | **0.013** |
| 2011 | **.9276** | **.1398** | **-0.50** | **0.619** |
| 2012 | **1.3900** | **.2258** | **2.03** | **0.043** |
| 2013 | **1.2901** | **.2034** | **1.62** | **0.106** |
| 2014 | **1.4228** | **.2319** | **2.16** | **0.030** |
| 2015 | **1.4625** | **.2262** | **2.46** | **0.014** |
| 2016 | **1.5144** | **.2727** | **2.30** | **0.021** |
| 2017 | **1.8251** | **.3092** | **3.55** | **0.000** |
| 2018 | **1.4119** | **.2357** | **2.07** | **0.039** |
| 2019 | **1.2650** | **.1965** | **1.51** | **0.130** |
| Year and Resident in Scotland (relative to 1998 and living in England/Wales) | | | | |
| 1999 | **.9925** | **.0813** | **-0.09** | **0.927** |
| 2000 | **1.4816** | **.1268** | **4.59** | **0.000** |
| 2001 | **.9793** | **.0910** | **-0.22** | **0.822** |
| 2002 | **1.0050** | **.0920** | **0.06** | **0.956** |
| 2003 | **.8582** | **.0784** | **-1.67** | **0.095** |
| 2004 | **.4836** | **.0379** | **-9.26** | **0.000** |
| 2005 | **.5742** | **.0454** | **-7.01** | **0.000** |
| 2006 | **.4790** | **.0412** | **-8.55** | **0.000** |
| 2007 | **.6127** | **.0510** | **-5.88** | **0.000** |
| 2008 | **.5177** | **.0416** | **-8.19** | **0.000** |
| 2009 | **.7382** | **.0598** | **-3.74** | **0.000** |
| 2010 | **.8640** | **.0735** | **-1.72** | **0.086** |
| 2011 | **1.2406** | **.1628** | **1.64** | **0.100** |
| 2012 | **1.2976** | **.1728** | **1.96** | **0.051** |
| 2013 | **1.4279** | **.1950** | **2.61** | **0.009** |
| 2014 | **1.2373** | **.1602** | **1.64** | **0.100** |
| 2015 | **1.5615** | **.2110** | **3.30** | **0.001** |
| 2016 | **1.9798** | **.2865** | **4.72** | **0.000** |
| 2017 | **1.3033** | **.1624** | **2.13** | **0.034** |
| 2018 | **1.6655** | **.2342** | **3.63** | **0.000** |
| 2019 | **2.3262** | **.3511** | **5.59** | **0.000** |
|  |  |  |  |  |
| Constant | **3.7641** | **.3211** | **15.53** | **0.000** |
|  |  |  |  |  |

**Wald chi2(52) = 1533.49 (p<0.01); N = 37,238**

**
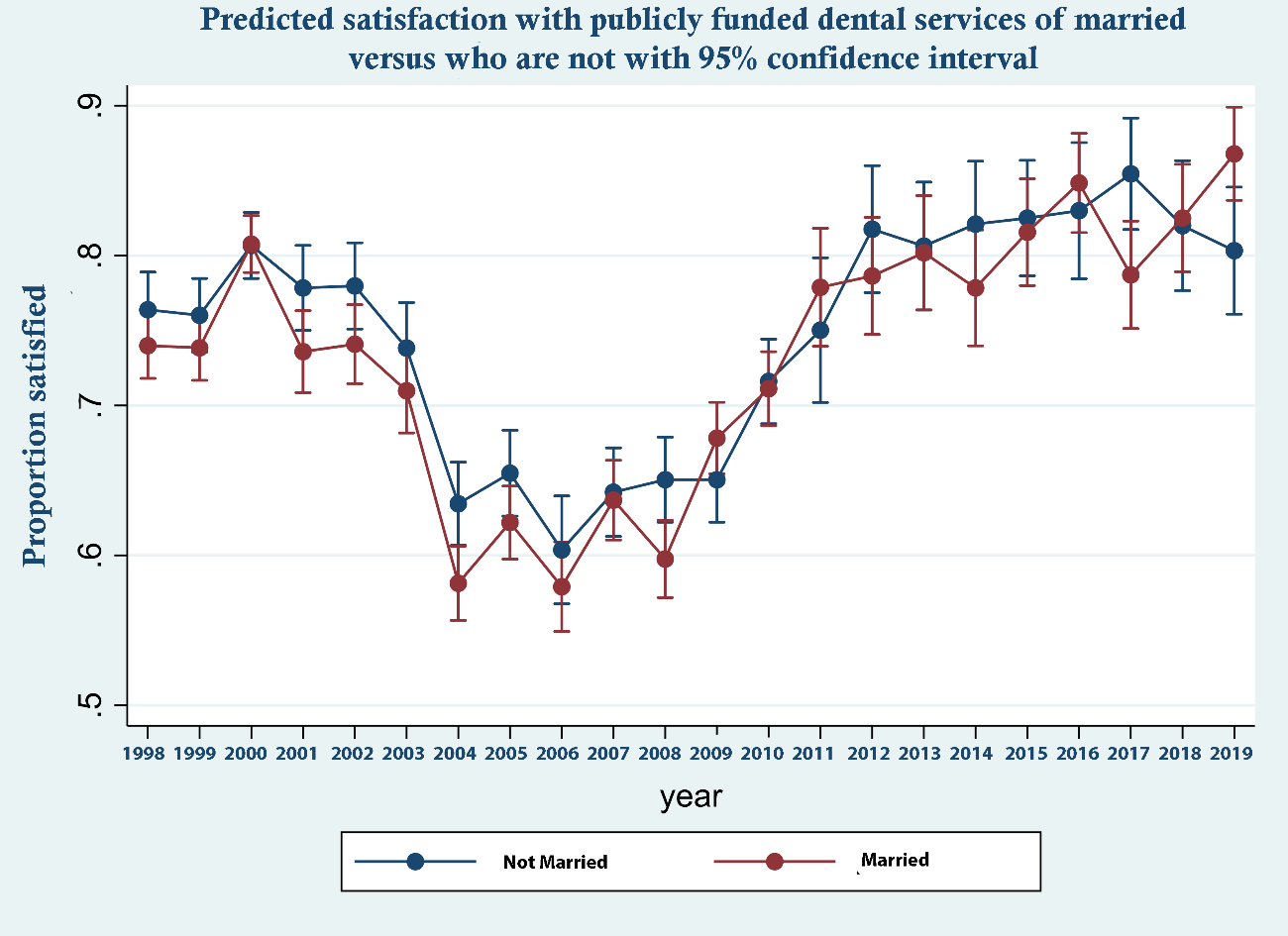
**

**Figure F8: Predicted satisfaction with publicly funded dental services of married versus who are not.**

**( unweighted )**
