# Supplementary material for: Satisfaction with dental care services in Great Britain 1998–2019
Source: BMC Oral Health. 2022 Jul 26;22:308. doi: 10.1186/s12903-022-02343-7 (PMC9315088; doi:10.1186/s12903-022-02343-7)
Supplement: Supplementary file 4 — Additional file 4. Weighted analysis in where neither satisfied nor dissatisfied is grouped with dissatisfied groups. [file 12903_2022_2343_MOESM4_ESM.docx]

**Online Supplement 4**

**Table A1 : Logistic regression of satisfaction ( satisfied or very satisfied versus neither satisfied nor dissatisfied, dissatisfied and very dissatisfied) with NHS dental services.**

| **Independent variable** | **Odds Ratio** | **Std. Err.** | **z** | **P>\|z\|** | **[95% Conf. Interval]** | |
| --- | --- | --- | --- | --- | --- | --- |
|  |  |  |  |  |  | |
| **Had Dependent Child in Household** | **1.2040** | **.0321** | **6.95** | **0.000** | **1.1425** | **1.2688** |
| **Had Degree** | **.7457** | **.0243** | **-9.00** | **0.000** | **.6995** | **.7949** |
| **Married** | **1.0091** | **.0275** | **0.33** | **0.739** | **.9565** | **1.0646** |
|  |  |  |  |  |  |  |
| **Income Quartile (relative to 1)** | | | | | | |
| **2** | **.9806** | **.0336** | **-0.57** | **0.569** | **.9169** | **1.0487** |
| **3** | **.8872** | **.0326** | **-3.26** | **0.001** | **.8255** | **.9535** |
| **4** | **.7377** | **.0294** | **-7.62** | **0.000** | **.6822** | **.7977** |
|  |  |  |  |  |  |  |
| **Male** | **.8125** | **.0193** | **-8.71** | **0.000** | **.7754** | **.8513** |
| **Resides in Scotland** | **1.5178** | **.0630** | **10.05** | **0.000** | **1.3992** | **1.6465** |
| **White** | **1.0976** | **.0504** | **2.03** | **0.042** | **1.0031** | **1.2010** |
| **Over65** | **1.0507** | **.0339** | **1.53** | **0.126** | **.9862** | **1.1195** |
|  |  |  |  |  |  |  |
| **Year (relative to 1998)** | | | | | | |
| **1999** | **1.0344** | **.0621** | **0.56** | **0.573** | **.9195** | **1.1638** |
| **2000** | **1.4499** | **.0880** | **6.12** | **0.000** | **1.2873** | **1.6330** |
| **2001** | **1.0675** | **.0722** | **0.97** | **0.334** | **.9349** | **1.2189** |
| **2002** | **1.0455** | **.0702** | **0.66** | **0.507** | **.9165** | **1.1928** |
| **2003** | **.98101** | **.0657** | **-0.29** | **0.775** | **.8603** | **1.1186** |
| **2004** | **.6190** | **.0374** | **-7.92** | **0.000** | **.5498** | **.6970** |
| **2005** | **.7120** | **.0436** | **-5.55** | **0.000** | **.6314** | **.8028** |
| **2006** | **.5881** | **.0405** | **-7.70** | **0.000** | **.5138** | **.6732** |
| **2007** | **.6455** | **.0412** | **-6.85** | **0.000** | **.5696** | **.7316** |
| **2008** | **.6512** | **.0403** | **-6.91** | **0.000** | **.5767** | **.7354** |
| **2009** | **.8072** | **.0497** | **-3.47** | **0.001** | **.7153** | **.9108** |
| **2010** | **.9001** | **.0577** | **-1.64** | **0.101** | **.7938** | **1.0206** |
| **2011** | **1.1069** | **.1043** | **1.08** | **0.281** | **.9200** | **1.3316** |
| **2012** | **1.2894** | **.1253** | **2.62** | **0.009** | **1.0657** | **1.5600** |
| **2013** | **1.2502** | **.1189** | **2.35** | **0.019** | **1.0376** | **1.5065** |
| **2014** | **1.0609** | **.0980** | **0.64** | **0.522** | **.8851** | **1.2716** |
| **2015** | **1.1584** | **.1062** | **1.60** | **0.109** | **.9678** | **1.3865** |
| **2016** | **1.4860** | **.1438** | **4.09** | **0.000** | **1.2293** | **1.7963** |
| **2017** | **1.2311** | **.1121** | **2.28** | **0.022** | **1.0298** | **1.4718** |
| **2018** | **1.4268** | **.1420** | **3.57** | **0.000** | **1.1739** | **1.7342** |
| **2019** | **1.4799** | **.1405** | **4.13** | **0.000** | **1.2285** | **1.7828** |
|  |  |  |  |  |  |  |
| **Constant** | **1.3299** | **.0879** | **4.31** | **0.000** | **1.1683** | **1.5139** |

**Wald chi2(31) =1079.91 (p<0.01) N = 37,238**


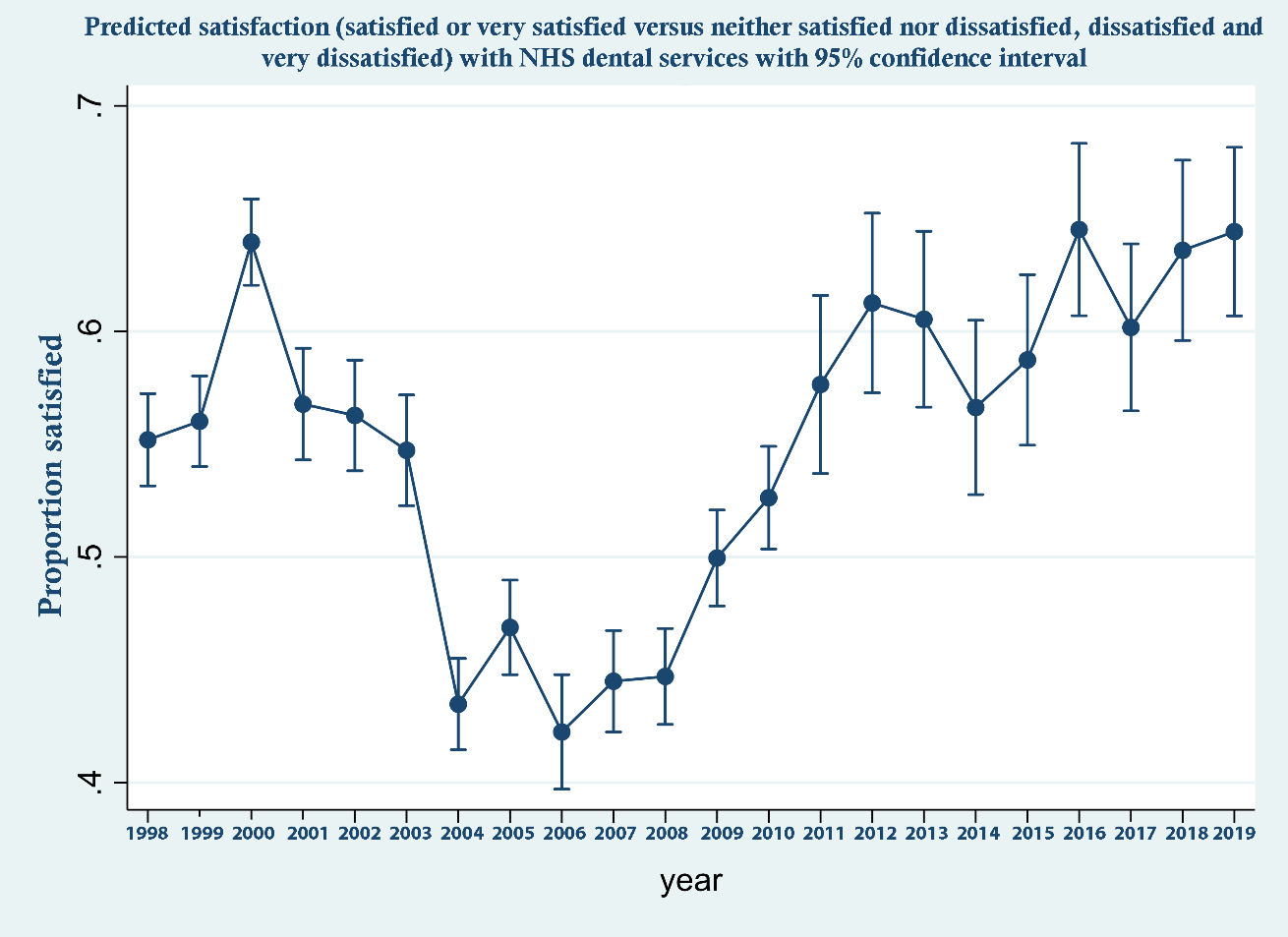


**Figure A1: Predicted satisfaction (satisfied or very satisfied versus neither satisfied nor dissatisfied, dissatisfied and very dissatisfied) with NHS dental services.**
